# An Overview of Mucosa-Associated Protozoa: Challenges in Chemotherapy and Future Perspectives

**DOI:** 10.3389/fcimb.2022.860442

**Published:** 2022-04-25

**Authors:** Helena Lucia Carneiro Santos, Karina M. Rebello

**Affiliations:** Laboratório de Estudos Integrados em Protozoologia, Instituto Oswaldo Cruz, Fundação Oswaldo Cruz (FIOCRUZ), Rio de Janeiro, Brazil

**Keywords:** Entamoeba histolytica, Giardia lamblia, Cryptosporidium spp., Trichomonas vaginalis, reinfection, treatment-refractory, repurposed drug

## Abstract

Parasitic infections caused by protozoans that infect the mucosal surfaces are widely neglected worldwide. Collectively, *Entamoeba histolytica, Giardia lamblia, Cryptosporidium* spp. and *Trichomonas vaginalis* infect more than a billion people in the world, being a public health problem mainly in developing countries. However, the exact incidence and prevalence data depend on the population examined. These parasites ultimately cause pathologies that culminate in liver abscesses, malabsorption syndrome, vaginitis, and urethritis, respectively. Despite this, the antimicrobial agents currently used to treat these diseases are limited and often associated with adverse side effects and refractory cases due to the development of resistant parasites. The paucity of drug treatments, absence of vaccines and increasing problems of drug resistance are major concerns for their control and eradication. Herein, potential candidates are reviewed with the overall aim of determining the knowledge gaps and suggest future perspectives for research. This review focuses on this public health problem and focuses on the progress of drug repositioning as a potential strategy for the treatment of mucosal parasites.

## 1 Introduction

Pathogenic protozoa associated with mucosal surfaces represent a significant threat to global health and development. Their control and elimination rely on prevention, diagnosis, and effective treatment. Altogether, *Entamoeba histolytica, Cryptosporidium* spp., *Giardia intestinalis* (syn. *Giardia lamblia, Giardia duodenalis*) contribute significantly to the global burden of gastroenteritis and fit well into the One Health concept (Collaborators, 2017; Shrivastav et al., 2020; Li et al., 2021). In turn, *Trichomonas vaginalis* accounts for one of the most prevalent non-viral sexually transmitted infections (STIs) (Vivancos et al., 2018). However, to a lesser extent, sexual activity that promotes faecal-oral contact can also lead to the transmission of *E. histolytica*, *Cryptosporidium* spp., and *G. intestinalis*. These intestinal infections of interest within the STI scope pose an important public health challenge and seem to be significantly underestimated (Hung et al., 2012; Escobedo et al., 2014; Billet et al., 2019).

Globally, these mucosa-associated protozoa are one of the leading causes of morbidity and mortality, mainly in areas of intense poverty, in marginal communities of urban centres and rural areas in developing countries (Iyer et al., 2017). The clinical spectrum of diseases produced by these protozoa ranges from asymptomatic (up to 50%) to severe disease, which includes liver abscesses, malabsorption syndrome, vaginitis, and urethritis (Collaborators, 2017; Leung et al., 2019; Li et al., 2021). Moreover, despite a low frequency, trichomoniasis has been implicated in leading to serious adverse effects, such as infertility and cervical cancer (Yang et al., 2018). In pregnancy, *T. vaginalis* is frequently linked with complications, such as premature birth and low birth weight babies (Silver et al., 2014; Leitsch, 2016). While in men, symptomatic infections are rarer (urethritis and prostatitis), but in the long term, trichomoniasis may lead to impaired sperm quality (Mielczarek and Blaszkowska, 2016) and an increased risk of acquisition and transmission of human immunodeficiency virus (HIV) and other STIs (Kissinger and Adamski, 2013; Lazenby et al., 2019) in both sexes.

Remarkably, despite the good and high coverage of sanitary quality, these parasites are still a matter of concern to developed countries (e.g., in European countries and the North American states) (Nisha et al., 2013; Gharpure et al., 2019). The outbreaks seem to be concentrated in the USA, Canada, Australia and the UK (Karanis et al., 2007; Painter et al., 2016). Large outbreaks are associated with treated (disinfected) recreational water venues in the USA. In the last decade, *Cryptosporidium* has emerged as one of the major causes of outbreaks associated with treated aquatic venues. Each year, in the USA, 748,000 cases of cryptosporidiosis (Scallan et al., 2011) and more than 1.2 million cases of *G. intestinalis* are diagnosed (Collier et al., 2021). These parasites are zoonotic agents that are often identified during outbreaks caused by contaminated public water supplies (Nisha et al., 2013; Painter et al., 2015). It is noteworthy that outbreaks of cryptosporidiosis have been related to public drinking water (failures at water treatment facilities), as in the massive Milwaukee cryptosporidiosis outbreak that affected nearly 400,000 people (Mac Kenzie et al., 1994; Efstratiou et al., 2017). However, in some outbreaks, recreational, drinking and fountain waters have been identified as important sources of community infections worldwide (Karanis et al., 2007). As a result, children are more at risk for infection, and cryptosporidiosis is more prevalent in children and immunosuppressed patients. Similarly, children are more likely to have giardiasis and amebiasis than adults (Iyer et al., 2017).

In regard to *T. vaginalis*, in the USA it is one of the most common causes of protozoal infections (Satterwhite et al., 2013; Secor et al., 2014). There were an estimated 2.6 million infections in 2018, and it is also a common cause of symptomatic vaginitis in women (Newman et al., 2015; Hlavsa et al., 2017; Conners et al., 2021). Globally, trichomoniasis mostly affects women between 35 and 40 years of age (Hung et al., 2012; Escobedo et al., 2014).

## 2 Treatment and Side Effects

For many years, some pathogenic protozoa parasites associated with mucosal surfaces were considered “neglected infections of poverty”, had low visibility and their studies were unable to attract funding, leading to insufficient development of their diagnosis and treatment options. Currently, they are underdiagnosed, and due to the lack of an effective vaccine, chemotherapy is the only option against these infections.

There are only a small number of drugs available that are effective against these microorganisms. Noteworthy, with different availability across countries, basically two classes of drugs are used in treatment-the nitroimidazole derivatives (MTZ, tinidazole, secnidazole and ornidazole) and benzimidazole derivatives (albendazole and mebendazole), Nitazoxanide, furazolidone, quinacrine, chloroquine and paromomycin. Consequently, rational use of available antiparasitic agents is essential to maintain their usefulness. Although, the cure rate is great, treatment failure can be owing to several factors including noncompliance, resistance and reinfection.

A non-exhaustive list of treatments selected from the literature is depicted in **Table 1**.

**Table 1:** Current treatment options for amoebiasis, giardiasis, cryptosporidiosis and trichomoniasis.

Diseases	Parasite	Drug	References
Amoebiasis	*E. histolytica*	Nitroimidazoles (Metronidazole, tinidazole, ornidazole, secnidazole), Emetine, Paromomycin Nitazoxanide	(Shirley et al., 2018; Gonzales et al., 2019; Sharma and Ahuja, 2021)
Giardiasis	*G. intestinalis*	Nitroimidazoles (Mebendazole and Tinidazole) Benzimidazole (Albendazole and Mebendazole) Furazolidone, Paromomycin nitazoxanide	(Morgan et al., 1993; Rossignol, 2010; Kappagoda et al., 2011; Vivancos et al., 2018)
Trichomoniasis	*T. vaginalis*	Nitroimidazoles (Metronidazole and Tinidazole)	(Gulmezoglu and Garner, 1998; Meites et al., 2015; Sherrard et al., 2018; Workowski et al., 2021)
Cryptosporidiosis	*Cryptosporidium* spp.	Nitazoxanide	(Rossignol, 2010; Innes et al., 2020)

Some of the current pharmacological treatments of amebiasis, giardiasis, cryptosporidiosis, and trichomoniasis are discussed in more detail. The most effective and widely used compound is MTZ (a nitroimidazole derivative) that has been the mainstay of protozoan parasite treatment for decades (Leitsch, 2019).

This compound has the ability to reduce and produce nitro radicals in anaerobic microorganisms that inhibit pathogen DNA synthesis (Leitsch, 2019). Events associated with MTZ therapy, such as drug adverse effects or the need for continued dosing past the resolution of disease symptoms, are common (Hernandez Ceruelos et al., 2019). In addition, there is emerging evidence for an increased frequency of therapeutic failure (Leitsch, 2015). In spite of this, the most used medication against these protozoa, except for *Cryptosporidium* spp., is still MTZ. Although its mechanism of action is not fully elucidated, MTZ is activated by thioredoxin reductase and possibly by ferredoxin to generate a nitro-radical anion and a nitroimidazole compound on subsequent reduction. These metabolites compete with the energy metabolism pathway of anaerobic microorganisms, causing toxic effects on trophozoites by inhibiting nucleic acid synthesis, thereby inhibiting protein synthesis and parasite growth (Shrivastav et al., 2020; Weir and Le, 2021). MTZ has been prescribed for over 60 years for luminal and tissue action on *G. intestinalis* and *T. vaginalis*, respectively. Moreover, this drug is currently the standard therapy for treating adults and children with invasive amebiasis. However, treatment for amebiasis is reliant on a single class of agents, the nitroimidazole compounds (Haque et al., 2003). Advantages of MTZ are that it is effective at killing trophozoites, cheap, and can be orally dosed. However, it is unable to kill the infective cyst stage of *E. histolytica* from the colonic lumen, necessitating a multi-drug treatment regimen. Consequently, around 40% of patients treated with MTZ will continue to have parasites in the colonic lumen (Haque et al., 2003) and a second agent (paromomycin or iodoquinol) should be administrated to completely clear the remaining trophozoites and cysts from the colonic lumen. Other unwanted side effects include alcohol intolerance and problems with use during pregnancy and lactation (Roe, 1977).

Unfortunately, resistance to MTZ and tinidazole have been reported in *G. intestinalis* and *T. vaginalis* for several decades (Schwebke and Barrientes, 2006; Kirkcaldy et al., 2012; Paulish-Miller et al., 2014; Leitsch, 2015). Several investigations have identified MTZ-resistant *T. vaginalis* strains associated with treatment-refractory vaginal trichomoniasis (Coelho, 1997; Meri et al., 2000; Schmid et al., 2001; Lin et al., 2020). However, nitroimidazole resistance is relative not absolute, ranging from slight to strong. Similar findings were reported for refractory giardiasis due to MTZ resistance in the parasite in both healthy and immunocompromised individuals (Morch et al., 2008; Nabarro et al., 2015; Carter et al., 2018; Lalle and Hanevik, 2018). So far, there is slight evidence for MTZ resistance in *E. histolytica* (Victoria-Hernandez et al., 2020), but a decrease in 5-nitroimidazole susceptibility can be induced experimentally (Wassmann et al., 1999; Iyer et al., 2017). Notoriously, increased drug resistance leads to higher doses used in the treatment and, therefore, more severe side effects (Hernandez Ceruelos (Hernandez Ceruelos et al., 2019). MTZ is generally well tolerated, but unpleasant side effects are often reported. The most common adverse effects are dose dependent, mild and reversible. Among them, common secondary effects of the nitroimidazoles are gastrointestinal tract symptoms, such as nausea, anorexia, vomiting and metallic or bitter taste, dizziness, ataxia and headache (Hernandez Ceruelos et al., 2019; Diptyanusa and Sari, 2021).

It is also important to note that the risk of treatment failure is linked to reinfection, suboptimal drug dose and drug resistance. In particular, *G. intestinalis* could be sequestrated in the gallbladder or the pancreatic duct, which promotes treatment failure (Escobedo et al., 2016; Lalle and Hanevik, 2018). In turn, except for *T. vaginalis*, it is hard to make this distinction in an endemic area, because reinfection is frequent related to high environmental contamination by infective cysts/oocysts, in association with precarious sanitation conditions. However, treatment failure must be excluded in the case of a recurrence of symptoms after appropriate therapy.

Nitazoxanide (NTZ) is a nitrothiazole pro-drug that also needs to be reduced at its nitro group to be active. NTZ has been shown to be a non-competitive inhibitor of the pyruvate:ferredoxin/flavodoxin oxidoreductases (PFORs) of *T. vaginalis*, *E. histolytica* and *G. intestinalis* (Adagu et al., 2002). Protein disulphide isomerases (PDI) have also been identified as potential targets of NTZ activity. In *G. intestinalis*, which expresses PDI variants, PDI2 and PDI4 expression was shown to be significantly downregulated during *in vitro* treatment with NTZ, indicating that the drug is targeting this enzyme (Muller et al., 2007).

In general, the effects of NTZ on the trophozoite ultrastructure of *T. vaginalis*, *E. histolytica* and *G. intestinalis* include cell swelling and distorted cell shape, a redistribution of vacuoles, plasma membrane damage and the formation of extensive empty areas in the cytoplasm of the protozoa (Cedillo-Rivera et al., 2002). Conversely, NTZ is currently the single approved medicine for the treatment of cryptosporidiosis in individuals who have healthy immune systems. It exhibits moderate clinical efficacy in children and immunocompromised individuals, although the treatment of immunosuppressed patients is remarkably difficult, even with higher doses (Abubakar et al., 2007; Amadi et al., 2009). Common side effects may include: nausea, diarrhoea, dry mouth, skin rash, stomach pain, fatigue, headache, and scleral and urine discoloration (Andersson, 1981; Diptyanusa and Sari, 2021).

## 3 Prospects About of Mucosa-Associated Protozoa Infections Persistence: A Challenge Ahead

Mucosa-associated protozoa infections have distinct characteristics that, when considered together, set them apart from other diseases. Of note, the persistence of infection is linked to several causes, including suboptimal drug concentrations, incomplete treatment regimens, reinfection, an immunocompromised state and drug resistance. Understanding of these factors is, thus, relevant and has been demonstrated. With mass drug administration (MDA), continued campaigns have substantially reduced soil-transmitted helminth (STH) infections in many countries. Albendazole is frequently used in MDA campaigns as a principal control tool for intestinal helminth infections. The collateral benefits of preventive chemotherapy might also affect conditions beyond its originally intended targets, such as enteric protozoa like *G. lamblia*, *E. histolytica* and *Cryptosporidium* spp. However, the therapeutic regimen of treatment with albendazole is suboptimal for these protozoa and may lead to drug resistance (Quihui-Cota and Morales-Figueroa, 2012; Oliveira et al., 2020), although no surveillance system exists to detect resistance. In Pemba Island, Tanzania, persistent parasitic infection was reported following single-dose albendazole, NTZ and albendazole-nitazoxanide (Speich et al., 2013). Conversely, the rates of *Giardia*, *Cryptosporidium* and *E. histolytica* are high in communities where basic sanitary conditions are unsatisfactory or non-existent; consequently, the frequent episodes of reinfection and protozoan persistence are remarkable in these communities, even after treatment (Fantinatti et al., 2020). In turn, cases of recurrent trichomoniasis are likely to occur due to a lack of adherence to the medication or reinfection from an untreated sexual partner (Sena et al., 2014). However, decreased sensitivity to nitroimidazoles has been identified in 2% to 10% of vaginal trichomoniasis cases (Perez et al., 2001; Schwebke and Barrientes, 2006; Kirkcaldy et al., 2012).

Notably, there are important challenges in the context of these infections. The first challenge is the lack of reliable epidemiological data, which precludes one from estimating the actual burden of pathogenic protozoa associated with mucosal surfaces. However, the heart of this challenge is mainly associated with two (some) factors: a high percentage of asymptomatic individuals and limited tests for diagnosis, which are biased and likely contribute to the epidemiological data underestimating the actual global burden of these diseases (Soares et al., 2020).

The second challenge are factors intrinsic to the host and/or parasite. The complex parasite-host relationship involves multiple mechanisms that have been characterized as one of the most important challenges of these times. Despite being complex and poorly investigated, the existence of one organism within another is not uncommon between protists and bacteria and/or viruses. There is an increasing recognition that endosymbionts in protozoa could influence the outcome of a disease as an eco-evolutionary process, such as drug resistance (Barrow et al., 2020). Parasites infected by viruses modify this relationship, adding more complexity to the system that now becomes tripartite. However, these issues remain largely unknown and evidence is conflicting.

Globally, some strains of *T. vaginalis, G. intestinalis, E. histolytica*, and *Cryptosporidium* spp. carry endosymbiotic double-stranded RNA (dsRNA) viruses (Banik et al., 2014; Jenkins et al., 2015), which share common characteristics, with uncharted implications to both the human host and protozoa (Fichorova et al., 2012). However, increasing evidence is accumulating that these protozoa harbour different classes of viruses that are absent from humans, but their role in pathogenicity is still poorly understood. Indeed, much of our understanding of viral endosymbionts of mucosa-associated protozoa is based on the *Trichomonasvirus* (TVV), which has been relatively more studied in some details than other protozoa. The TVV is a dsRNA virus that belongs to the Totiviridae family, which was described and characterized in the 1980s and has at least two core proteins, a capsid protein (CP) and an RNA-dependent RNA polymerase (RdRp). Currently, the TVV has been divided into four distinct viral strains (TVV1, TVV2, TVV3, and TVV4), ranging in size from 4.5 to 5 kbp, based on phylogenetic analyses and comparisons of genomic sequences (Su and Tai, 1996; Liu et al., 1998). The viral genome is never found free in the protozoan cell, and the positive strand viral transcripts synthesized within the viral particle by the CP/RdRp are translocated to the cell cytoplasm to be translated (Barrow et al., 2020). Despite their non-lytic life mode, dsRNA viruses may induce various phenotypic changes that may interfere with the virulence of *T. vaginalis* (Provenzano et al., 1997; Fraga et al., 2012). In the last few years, some studies have reported experimental findings that point toward a positive association between infection and the exacerbation of trichomoniasis symptoms (El-Gayar et al., 2016) while other authors have shown the absence of any correlation (El-Gayar et al., 2016; Graves et al., 2019). Of note, more than one TVV species can coexist in the same *T. vaginalis* cell (Benchimol et al., 2002), and a high percentage of Trichomonasvirus in different protozoan isolates have been reported worldwide, ranging from 30% to 100% (Fichorova et al., 2017), which may lead to the discrepancy of the findings. The implications of these co-infections so far are unclear, but evidence has shown that the TTV released from infected *T. vaginalis* cells induced inflammation upon treatment with metronidazole (MTZ) and may suppress host immune activation (Fichorova et al., 2012; Govender et al., 2020). Each viral strain affects different aspects of the parasite. TVV2 and TVV3 infections are strongly enfolded in the upregulation of the protozoan cysteine proteases (Provenzano et al., 1997), which are involved in modulating *T. vaginalis* cytoadherence to human host cells and in the degradation of the basement membrane, human cellular molecules, and secretory IgAs. While, TVV1 and TVV2 are related to the severity of clinical symptoms of trichomoniasis in humans (Fraga et al., 2012). Conversely, one study reported increased MTZ susceptibility in TVV-infected *T. vaginalis* (Malla et al., 2011), while another report observed drug resistance (Snipes et al., 2000). However, additional research is needed to clarify the effect of TVV infection on 5-nitroimidazole resistance in *T. vaginalis*.

Similar to TVV, *Giardiavirus* was first isolated from assemblage A of *G. intestinalis* (Wang and Wang, 1986) as a dsRNA virus and is a member of the family Totiviridae, which specifically infects trophozoites of the parasite *G. intestinalis*. The virus makes capsids that are released from the host to infect other cells, and when a high viral titre is observed, the growth of the parasite is suspended (Marucci et al., 2021). However, unlike *Leishmaniavirus* and *Trichomonasvirus*, the *Giardiavirus* does not seem to be linked with the virulence of the parasite (Gomez-Arreaza et al., 2017). Another *Giardiavirus* of the family *Totiviridae* is *G. canis* virus (GCV) (Cao et al., 2009), which was isolated from the *G. canis* strain. However, it is important to note that knowledge on *Giardiavirus* has unfortunately made little progress in recent years. Similarly, there are few reports on *Cryptosporidium* and *E. histolytica* virus. *Cryspovirus* is a new genus of protozoan viruses in the family Partitivirida, which infect different species of *Cryptosporidium* oocysts (Nibert et al., 2009). Although viruses can have complex effects on *Cryptosporidium*, there is so far no well-established information on whether the pathogenicity of parasites is either positively or negatively modulated by *Cryspovirus* infection (Banik et al., 2014; Sharma et al., 2016). Likewise, the presence of virus-like particles in an *E. histolytica* trophozoite has been reported. This virus was described in the 1960s based on electron microscopy studies, which revealed characteristics closely related to Rhabdoviridae (a family of negative-strand RNA viruses) (Ludvik and Shipstone, 1970). Despite being discovered a number of years ago, little was investigated about the viruses involved and their impact on amoebiasis, and no investigation based on clinical perspective has been done (Bird et al., 1974; Banik et al., 2014).

## 4 Parasite Drug-Resistance Mechanisms

The nitroimidazole metronidazole is clearly one of the best-studied drugs affecting intermediary metabolism in *Entamoeba histolytica*, *G. lamblia* and *Trichomonas vaginalis* (Lofmark et al., 2010), but resistance mechanisms against NTZ and albendazole were also investigated. Notoriously, Metronidazole’s mechanism of action describes that of the other nitroimidazoles. However, not all mechanisms that reduce susceptibility to the nitroimidazoles have been described. Resistance to metronidazole is complex but appears to be owing to activation of the prodrug to the active nitroso free radical. Laboratory-induced resistant isolates (Upcroft and Upcroft, 1993) is associated with an downregulation of pyruvate:flavodoxin/ferredoxin oxidoreductase (PFOR) activity, a protein absenting in higher eukaryotic cells (Horner et al., 1999). Metronidazole is a prodrug that present an ability to intracellularly reduce to intermediates nitro radical (Sisson et al., 2000) by electrons coming from PFOR (Upcroft and Upcroft, 2001). This radical causes irreversible damage on intracellular structures of parasites. Some enzymatic pathways of *G. lamblia* and *Trichomonas vaginalis*, were identified that are probable to play a function in 5-nitroimidazole reduction, inclusive of the central metabolic enzyme pyruvate:ferredoxin oxidoreductase (PFOR) together with ferredoxin (Townson et al., 1996; Rasoloson et al., 2002; Leitsch et al., 2011) and thioredoxin reductase (TrxR).

In Giardia, pyruvate:ferredoxin oxidoreductases (PFORs), Giardia lamblia nitroreductase 1 (GlNR1), and thioredoxin reductase (TrxR) are capable to induce the partial reduction reaction that to convert MTZ into toxic metabolites (Nillius et al., 2011; Lalle and Hanevik, 2018).

It is worth to mention that large predictor studies for therapeutic failure are unavailable so far. The resistance slowly induced in laboratory lines could be of a different nature than the rapidly increasing clinical resistance occurred the past decades. Laboratory-induced resistance were obtained from patients several decades ago, most of them belong to the zoonotic assemblage AI, and may not currently represent the main circulating strains (Lalle and Hanevik, 2018). The results reported from laboratory-induced resistant lines showed a variation in the infectivity and molecular phenotypes, suggesting that multiple molecular resistance phenotypes are possible. However, stability of laboratory-induced resistance is generally lost or reduced after removing the drug. MTZ resistances in clinical isolates from patients were demonstrated in a study reported that some isolates were also less MTZ susceptible when tested in a neonatal mouse model (Lemee et al., 2000). Studies with metronidazole-resistant strains have reported, however, that resistance is not always correlated with reduced POR activity (Leitsch, 2015). A study on resistance of clinical isolates of *T. vaginalis* reported that flavin reductase activity was downregulated (Leitsch et al., 2012), or even absent, in metronidazole-resistant strains. It is important to considerate that flavin reductase can reduce oxygen to hydrogen peroxide (Leitsch et al., 2014), so its downregulation might impair oxygen scavenging, and with activation of nitroimidazoles by either inhibiting drug-activating pathways or by reoxidizing a critical, toxic, nitroradical anion intermediate, consequently reduce metronidazole uptake. Likewise, resistance to metronidazole in *E. histolytica* has been associated with oxidative stress mechanisms, including superoxide dismutase and peroxiredoxin, without a significant decrease of the PFOR activity (Wassmann et al., 1999).

NTZ resistance is thought to be due to altered expression of stress response proteins. An expression study on the NTZ laboratory-induced resistance showed several protein chaperones (Hsp70, Hsp90, and Cpn60), (Leitsch, 2015) and surface antigens to be upregulated in expression (Muller et al., 2008). Levels of PFOR were practically unaltered in nitazoxanide-resistant cell lines (Muller et al., 2007), whereas nitroreductase 1 was found downregulated (Nillius et al., 2011). It is important to point out that resistance to NTZ has been reported not only on experimentally induced resistant strains, but also in clinical isolates (Lemee et al., 2000; Reyes-Vivas et al., 2014).

Benzimidazole affect the microtubules assembly, and induction of oxidative stress may also play a role in the antiparasitic mechanism. Albendazole resistance in *Giardia* is correlated with cytoskeletal changes (Upcroft et al., 1996), in the particular emphasis on the median body. Moreover, resistance has been attributable mutation in the beta-giardin gene, leading in amino acid changes have also been associated with reduced susceptibility to albendazole (Jimenez-Cardoso et al., 2009). However, ttreatment failure has also been reported for ABZ either administered alone or in combination with MTZ. The higher levels of efficacy with albendazole require multiple doses.

In fact, given their significant impact on human health and infections of mucosa-associated protozoa being difficult to eradicate, it is necessary to focus on finding preventive and curative therapeutics to control their spread. Thereby, there is an urgent need to develop new therapeutic options that will help fight resistance and increase effectiveness. However, a great barrier is the high costs associated with developing a new drug and low economic returns (Dimasi et al., 2003). In recent years, one alternative approach for lowering the costs of drug discovery and development for these diseases is to repurpose drugs developed for other indications (Ashburn and Thor, 2004; Jourdan et al., 2020). It is an alternative viable to the identification of a new indication for a known drug or compound with previously established preclinical studies or clinical data can significantly decrease the overall cost and reduce the time required to bring a drug to market (Muller and Hemphill, 2013).

Recent strategies for the development of drugs in the context of these mucosa-associated parasitic protozoa will be discussed in the next section.

## 5 Recent Approaches to Drug Development

Over the past six decades, the lack of financial incentives has strongly reduced efforts to develop effective treatments. Because of this, new drugs are urgently required for parasitic protozoan infections, and repurposing drugs is a promising approach for the drug development process, a rapid mode and reduces cost. The discovery of new uses for approved drugs has the potential to identify and reveal new targets that aim to maximize the pre-existing preclinical and clinical knowledge accumulated on registered drugs for a new indication outside the scope of its original indication. Thus, it opens a promising avenue for identifying targets for treatment.

Nevertheless, the treatment of parasitic diseases is often complex, owing to the multiple life stages of parasites with differing sensitivities to chemical agents. High-throughput screens of repurposed drug libraries have been used to the screening of drugs for *E. histolytica, G. intestinalis, Cryptosporidium* spp. and *T. vaginalis*, and some of these compounds are shown below. However, one of the available practical approaches to find novel potential candidates with antiprotozoal activity are FDA-approved drugs, which is an attractive way to identify new drugs.

### 5.1 Advances in Research for Novel Anti-*Giardia* Activity Agents and FDA-Approved Drugs

#### 5.1.1 Fumagilin

This is an antibiotic compound isolated from *Aspergillus fumigatus*, and its efficacy has been demonstrated in the treatment of diarrheal disease caused by *Microsporodia* spp. (Goodgame, 2003; Agholi et al., 2013). Similarly, fumagillin also showed activity against *Giardia* assemblages A and B at submicromolar concentrations in the mouse giardiasis model. The antigiardiasic properties of fumagilin have exhibited effectiveness, even against *in vitro* MTZ resistance, and the *in vivo* animal model was superior to MTZ, being a promising drug candidate for the treatment of giardiasis (Kulakova et al., 2014).

#### 5.1.2 Proton Pump Inhibitors

PPIs are a class of medications widely used for the treatment of gastroesophageal reflux disease and other acid-related disorders. Repurposing of PPIs, such as omeprazole, is effective *in vitro* against *G. lamblia*, and the toxic activity is associated with the inhibition of triosephosphate isomerase (*Gl*TIM), which is a key enzyme in glucose and glycogen metabolism of the parasite (Reyes-Vivas et al., 2014; Lopez-Velazquez et al., 2019). Hernández-Ochoa and colleagues (2017) described two novel PPI derivatives, named BHO2 and BHO3, that showed better antigiardiasic activity than omeprazole in micromolar concentrations, without a cytotoxic effect on mammal cell cultures (Hernandez-Ochoa et al., 2017; Hernandez-Ochoa et al., 2020).

#### 5.1.3 Tetrahydrolipstatin (orlistat)

This is a specific lipase inhibitor derived from lipstatin, which is a lipid produced by *Streptomyces toxytricini* used in the treatment of obesity. Hahn and colleagues (2013) showed experimentally that orlistat inhibited *in vitro* growth of *G. duodenalis* at low micromolar concentrations compared to that of the standard drug MTZ (Hahn et al., 2013).

#### 5.1.4 Spiro Compounds of Isatin

Spiro compounds represent an important class of a small and versatile organic molecule that are known to exhibit versatile biological properties. *In vitro* test of 1*H*-1,2,3-triazole and β-amino-alcohol tethered isatin-β-lactam conjugates displayed anti−*T*. *vaginalis* activity (Nisha et al., 2013). Also, 1*H*-1,2,3-triazole-tethered isatin-MTZ conjugates also exhibited activity *in vitro* against *T. vaginalis, E. histolytica*, and *G. lamblia* in submicromolar concentrations (Kumar et al., 2018).

#### 5.1.5 Auranofin

The gold(I) complex auranofin was approved by the FDA in 1985 as an oral anti-arthritic agent (used in the treatment of rheumatoid arthritis) was shown to be effective against MTZ-resistant *Giardia*, and auranofin has now progressed into clinical trials (Capparelli et al., 2017).

#### 5.1.6 Disulfiram

This drug blocks an enzyme that is involved in metabolizing alcohol. It is an inhibitor of acetaldehyde dehydrogenase, which is used to support the treatment of alcohol use disorder. It also acts as a cysteine modifying agent. The antigiardiasic activity of disulfiram has shown to be effective against *G. lamblia* trophozoites *in vitro* and in a murine model of giardiasis. Interestingly, the drug has been demonstrated to be more than 2–4 times active against MTZ-resistant than MTZ-sensitive (WB and GS/B) parasites (Kulakova et al., 2014). This drug may act as a novel inactivator of *G. lamblia* triosephosphate isomerase (the endogenous activity and carbamate kinase *in vitro*), which demonstrates that its giardicidal effect may involve the inactivation of more than a single enzyme, increasing its potential as an antigiardial drug (Castillo-Villanueva et al., 2017).

### 5.2 Advances in Research for Novel Anti-*Entamoeba histolytica* Activity Agents and FDA-Approved Drugs

#### 5.2.1 Antineoplastic Kinase Inhibitors

Recently, antineoplastic kinase inhibitors have emerged as potent amebicidal drugs for amebiasis. The drugs tested were able to kill *E. histolytica* trophozoites as quickly as MTZ. Moreover, one of these drugs (ibrutinib) also exhibited both amebicidal and cysticidal properties, in contrast to all the drugs currently used in the therapeutic strategy (Sauvey et al., 2021).

#### 5.2.2 Rabeprazole

The thioredoxin system catalyses several redox reactions essential in maintaining a variety of important biological functions in the *Entamoeba*. It is considered a fundamental enzyme system (reducing the redox system and detoxifying the intracellular oxygen) for *E. histolytica* survival under both aerobic *in vitro* and *in vivo* conditions. Consequently, the TrxR/Trx system is an excellent target for antiamebic drug. Rabeprazole (RB), a drug widely used to treat heartburn, is able to inhibit the EhTrxR recombinant enzyme, amebic proliferation and several functions required for parasite virulence, such as cytotoxicity, oxygen reduction to hydrogen peroxide, erythrophagocytosis, proteolysis, and oxygen and complement resistances (Martinez-Perez et al., 2020).

#### 5.2.3 Pencolide

It is a fungal secondary metabolite that shows cysteine deprivation-dependent antiamebic activity. This compound targets cysteine synthase, which is essential for the proliferation and antioxidative defence of *E. histolytica* trophozoites, and is implicated in various important biological processes, including attachment, motility, proliferation, and antioxidative defence (Mori et al., 2018).

#### 5.2.4 Anisomycin and Prodigiosin

Anisomycin, an antibiotic isolated from *Streptomyces*, and prodigiosin, a natural red water-insoluble pigment isolated from *Serratia marcescens*, have already shown therapeutic effects over 50 years ago to treat amebiasis in small cohorts (Gonzalez Constandse, 1956; Ehrenkaufer et al., 2018). Ehrenkaufer and coworkers recently performed a screen of repurposed compounds against *E. histolytica*, anisomycin and prodigiosin were both able to kill MTZ-resistant parasites, while prodigiosin was active against mature cysts (Ehrenkaufer et al., 2018). This is the first compound showing efficacy against the dormant cyst form, which is highly resistant to environmental stresses and to the major drug used to treat amebiasis (MTZ).

Importantly, obatoclax an analogue of prodigiosin, which has been used in human clinical trials, had significant activity against both trophozoites and cysts. Development of this molecule as a therapeutic may be possible, given the established safety record in patients (Ehrenkaufer et al., 2020).

#### 5.2.5 Auranofin

It is a gold-containing compound originally developed to treat rheumatoid arthritis that showed antiparasitic activity against *E. histolytica* (Debnath et al., 2013; Andrade and Reed, 2015). This compound targets the thioredoxin reductase in *E. histolytica*, thereby making the parasite sensitive to oxidative stress (Debnath et al., 2012). It also showed 10-fold better activity against *E. histolytica* than the standard drug MTZ. Unsurprisingly, auranofin was equally active against *G. lamblia*, both in *in vitro* and *in vivo* studies (Debnath et al., 2013; Tejman-Yarden et al., 2013), indicating the possibility of its use for a more general treatment of protozoan parasites. However, it presented side effects, such as abdominal pain, nausea, anaemia, and elevated liver enzymes, and its use is prohibited during pregnancy.

#### 5.2.6 Disulfiram

This drug is an inexpensive orally administered drug used in the treatment of chronic alcoholism. It is rapidly metabolized to diethyldithiocarbamate (ditiocarb, DTC), which in the presence of zinc forms zinc diethyldithiocarbamate (ZnDTC). Gosh and colleagues demonstrated that the ZnDTC complex has a high *in vivo* activity against *E. histolytica* parasites (Ghosh et al., 2020). This metabolite of disulfiram showed the ability to inhibit COP9 signalosome, a critical upstream regulator of parasite protein degradation (Ghosh et al., 2020; Shirley et al., 2021).

### 5.3 Advances in Research for Novel Anti-*T. vaginalis* Activity Agents and FDA-Approved Drugs

#### 5.3.1 Ixazomib and Carmaphycin-17

The proteasome is a multicatalytic proteinase complex, and proteasome inhibitors have also been shown to be toxic for other pathogens and useful in parasitic treatment (Khare et al., 2016). O´Donoghue and colleagues (2019) validated the proteasome as a drug target for the development of a novel class of trichomonacidal agents (O’Donoghue et al., 2019). They showed experimentally that two clinically approved anticancer drugs, ixazomib and carmaphycin-17 (CP-17), were active against vaginal trichomonad infections. In addition, CP-17 was able to overcome MTZ resistance in *T. vaginalis* and had significant *in vitro* and *in vivo* efficacy against trichomonads (O’Donoghue et al., 2019).

#### 5.3.2 Benznidazole

In silico analyses classified 20 compounds as potentially active against *T. vaginalis*. From these, ipronidazole, dimetridazole and NTZ showed the highest cytocidal activity superior to MTZ and secnidazole, respectively. Besides, the *in vivo* assay revealed similar activity for benznidazole and MTZ, suggesting the former as a novel alternative in antitrichomonal therapy (Meneses-Marcel et al., 2018).

#### 5.3.3 Disulfiram

The antiprotozoal activities of disulfiram have been studied over the past decade. Disulfiram, when complexed to divalent metal ions (such as zinc) has been demonstrated as an anti-parasitic agent against protozoan parasites *Trypanosoma, Leishmania, Giardia* and *E. histolytica*. It also appears to be similarly effective against *T. vaginalis.*

This drug has shown antitrichomonal activity, and its metabolite, ditiocarb, has been shown to be more effective than MTZ against both sensitive and resistant trichomonads (Bouma et al., 1998).

### 5.4 Advances in Research for Novel Anti-*Cryptosporidium* Activity Agents and FDA-Approved Drugs

#### 5.4.1 FDA-Approved Drugs Phenotypic Screen

Even though promising targets and lead compounds have been identified, robust therapies to eliminate parasites are still lacking. Some of them (paromomycin, clarithromycin, azithromycin, rifaximin, rifabutin, and roxithromycin) (Shrivastava et al., 2017) have demonstrated limited potential when used in animal models, and all were ineffective in controlled trials in AIDS patients. Also, preclinical activity with miltefosine (originally developed as an anticancer drug) and clofazimine (leprosy drug) has been demonstrated, which had no efficacy in phase II studies in AIDS patients (Gavrilov, 1989; Croft et al., 2003; Sinkala et al., 2011; Huston, 2021).

Indeed, activity against an organism found by *in vitro* screening does not necessarily correlate to *in vivo* activity. Moreover, several aspects might explain unsuccessful drug discovery. One of the major bottlenecks to the development of specific anticryptosporidial drugs is the unavailability of a reproducible axenic *in vitro* culture system for *Cryptosporidium* spp., as well as being unable to genetically manipulate the organism.

The genomes of parasites have shown a limited biosynthetic capability that may be targets to identify promising new chemical entities. For example, there are approaches based on data mining of genome data to provide new insights into aspects of *Cryptosporidium* biology focused on novel targets. Recently, the identification of some ligands/inhibitors and parasite-specific molecules, such as parasite kinases; nucleic acid synthesis and processing; proteases; and lipid metabolism have paved the way for new therapies against *Cryptosporidium*, which is considered an attractive strategy. However, alternative salvage pathways could reduce the efficacy of many of these targets as one remarkable case demonstrated with the thymidylate synthase-dihydrofolate reductase target (Pawlowic et al., 2019)

#### 5.4.2 Bumped Kinase Inhibitors

Protein kinases play essential roles in the biology of *Cryptosporidium* and can identify essential pathways to potential new targets. Bumped kinase inhibitors (BKIs) targeting calcium-dependent protein kinase 1 (CDPK1) have shown effect against *Cryptosporidium in vitro* and in mice. Pyrazolopyrimidine analogues inhibit *C. parvum* CDPK1 and block *C. parvum* growth in tissue culture *in vitro*. However, the effect on parasite growth was variable and did not correlate well with enzyme inhibition (Kuhlenschmidt et al., 2016), conversely as demonstrated by *Toxoplasma gondii*. It also has shown issue challenges related to cardiovascular toxicity, teratogenicity, and varying efficacy (Choi et al., 2020; Love and Choy, 2021). Another series of BKI compounds have been developed which inhibit CDPK. However, this class of drugs has presented significant adverse reactions because of inhibited binding to mammalian protein kinases (Wang et al., 2020).

Another, kinase target is phosphatidylinositol-4-OH kinase, which phosphorylates lipid molecules to participate in intracellular signalling and trafficking, and is a target for pyrazolopyridines. Screening a library of compounds with antiparasitic activity found pyrazolopyridine KDU731 as a promising anticryptosporidial drug candidate [(Cryptosporidium lipid kinase PI(4)K (phosphatidylinositol-4-OH kinase)] that is active against both *C. parvum* and *C. hominis* (Manjunatha et al., 2017; Funkhouser-Jones et al., 2020). However, safety and pharmacological preclinical evaluations are necessary in order to support the initiation of human clinical trials.

#### 5.4.3 Inhibitors of Inosine Monophosphate Dehydrogenase

Another essential pathway in *Cryptosporidium* spp. is purine synthesis. Oxidoreductase inosine 5′-monophosphate dehydrogenase (IMPDH) is required for the conversion of adenosine into guanine nucleotides. IMPDH is a target for immunosuppressive, antiviral, and anticancer drugs. However, inhibitors of IMPDH have shown *in vitro* efficacy against *Cryptosporidium* spp (Jefferies et al., 2015).

#### 5.4.4 tRNA Synthetase

tRNA synthetases comprise a family of enzymes that couple specific amino acid residues to selected tRNA for protein peptide synthesis. *Cryptosporidium* spp. are inhibited by a benzoxaborole (a 3-aminomethyl benzoxaborole, AN6426) targeting leucyl-tRNA synthetase. Despite the fact that homologous proteins exist in humans, this molecule has the potential for antimicrobial drug design, since structural and sequence divergences can be modified to enhance specificity and avoid toxicity (Palencia et al., 2016). Besides this one, quinazoline-based derivative shows potent activity against *Cryptosporidium* prolyl-tRNA synthetase (Jain et al., 2017); imidazopyridine derivatives are also potent inhibitors of *Cryptosporidium* methionyl-tRNA synthetase (Buckner et al., 2019). Similar results have been observed with cladosporin derivatives, also exhibiting potent activity against *Cryptosporidium* lysyl-tRNA synthetases (KRSs) (Baragana et al., 2019).

#### 5.4.5 Cysteine Protease (Cryptopains)

Five genes coding cathepsin L-like proteases (cryptopains), a representative of clan CA, were identified in the *C. parvum* genome that are expressed during the sporozoite stage and are important for cell invasion and survival. The discovery that a cysteine protease inhibitor provides potent anticryptosporidial activity in an animal model of infection encourages the investigation and development of this class as a new (and urgently needed) therapy for cryptosporidiosis (Ndao et al., 2013).

## 6 Alternative Chemotherapeutic Agents Against Protozoan Parasites

### 6.1 Natural Compounds

Natural products remain an important source of biologically active substances and are an alternative source for parasitic control. In this context, several research groups are focused on the isolation and identification of novel compounds with antimicrobial activity from plant and fungal extracts, aiming to use them in the discovery of new antiprotozoal drugs. A list of natural compounds with antiprotozoal activity is described in **Supplementary Table 1**.

### 6.2 Hybrid Compounds

Studies have shown that hybrid molecules display high protozoal activity, making them potentially promising agents for antiprotozoal therapy. Evaluation of nitazoxanide–N-methylbenzimidazole hybrid compounds showed strong activity *in vitro* against *G. intestinalis*, *E. histolytica* and *T. vaginalis*, especially with *E. histolytica*, where the IC_50_ values ranged between 3 and 69 nM (Soria-Arteche et al., 2013).

Other compounds with antiprotozoal properties are 1H-1,2,3-triazole-tethered metronidazole-isatin conjugates, which presented inhibitory activity against *T. vaginalis*, *Tritrichononas foetus*, *G. lamblia* and *E. histolytica in vitro*. Curiously, compounds with thiosemicarbazone moieties showed better results against *G. lamblia* and *E. histolytica* and were similar to MTZ against trichomonads (Kumar et al., 2018). Matadamas-Martínez and colleagues (2016) showed that a nitazoxanide-N-methyl-1H-benzimidazole hybrid molecule exhibited giardicidal activity at nanomolar concentrations and was more active *in vitro* than both MTZ and albendazole, and equipotent to NTZ. This compound induced ultrastructural changes and alterations in cytoskeleton proteins and in proteins that play an important role in the encystment process (Matadamas-Martinez et al., 2016). Further *in vitro* and *in vivo* studies revealed that the hybrid compound also exhibited broad activity against susceptible and resistant strains to albendazole and NTZ (Matadamas-Martínez et al., 2020).

MTZ-chalcone conjugates exhibited activity against MTZ-susceptible and resistant strains of *T. vaginalis* (Anthwal et al., 2014). 3-(3,5-Difluoro-phenyl)-1-{4-[2-(2-methyl-5-nitro-imidazol-1-yl)-ethoxy]-phenyl}-propenone and 3-(3-chloro-phenyl)-1-{4-[2-(2-methyl-5-nitro-imidazol-1-yl)-ethoxy]-phenyl}-propenone compounds were four times more potent than MTZ and safe against HeLa *in vitro*, being a candidate drug capable of overcoming the MTZ resistance of *T. vaginalis* (Anthwal et al., 2014).

### 6.3 Synthetic Compounds

The *in vitro* antiprotozoal activities of 2H-indazole derivatives have been reported for *E. histolytica, G. lamblia* and *T. vaginalis* at lower concentrations and are usually more potent compared to MTZ, appearing to be a good alternative (Perez-Villanueva et al., 2017; Rodriguez-Villar et al., 2021). In turn, 2′-hydroxychalcone showed trichomonicidal *in vitro* analysis: at 12.5 µM associated with MTZ (40 µM), a reduction of 95.31% in the trophozoite’s viability was displayed after 24 hours of incubation (Das Neves et al., 2020). Furanyl *N*-acylhydrazone derivatives also presented activity against *T. vaginalis*, with IC_50_ values ranging from 1.69 µM to 1.98 µM (Alves et al., 2020).

MTZ thiosemicarbazones and ethyl- and methyl-quinoxaline-7-carboxylate 1,4-di-N-oxide derivatives showed a significant antiamoebic activity *in vitro*, with an IC_50_ of 0.56 µM and IC50 values ranging from 1.41 µM to 1.47 µM, respectively (Abid et al., 2008; Duque-Montano et al., 2013).

Muller and colleagues (2006) showed experimentally that NTZ-related thiazolide/thiadiazolide derivatives exhibited inhibitory activity *in vitro* against a *G. duodenalis* axenic culture and coculture with Caco2 cells (Muller et al., 2006). Another study revealed that 2-ethenyl- and 2-ethanyl-5-NI derivatives exhibited antigiardial activity without toxicity and were more potent that MTZ *in vitro*. Furthermore, they were more effective than MTZ in a murine giardiasis model (Valdez et al., 2009).

NTZ-related thiazolide/thiadiazolide compounds also exhibited *in vitro* inhibitory activity against *C. parvum.* Modifications of the NTZ chemical structure showed that anticryptosporidial activity is thought to be independent of the presence of a nitro group on the thiazole moiety, with IC_50_ lower than NTZ (Gargala et al., 2010).

Recently, *in vitro* and *in vivo* studies revealed that L-tert-leucyl thiazolide (aminoxadine), a soluble drug of tizoxanide (TIZ), possesses potency of cryptosporidiosis. *In vitro*, this compound dose*-*dependently inhibited *C. parvum* growth, and no toxicity was observed, since the IC_50_ for *C. parvum* (1.55 ± 0.21 μM) was at least 20-fold lower than the CC_50_ for HCT-8 cells. Surprisingly, in gerbils, a 5-day course of daily intramuscular aminoxanide treatment (100 mg/kg) resulted in a 72.5% oocyst excretion inhibition, statistically equivalent to 75.5% in rodents treated with a 4-fold lower oral dose of NTZ (Diawara et al., 2021). Until now, the only two injected drugs to inhibit *C. parvum* infection are aminoxanide and MMV665917, a new drug based on piperazine (Jumani et al., 2018).

### 6.4 Nanotechnology Against Protozoan Parasites

Nanotechnology-based drug delivery has emerged as a promising approach for several illnesses, including parasitic diseases, improving the ability to specifically target pathogens, penetrate barriers within the host to allow a drug to access areas of pathogen residence, reduce toxicity by lowering dose amount and frequency of administration, and increase the uptake of poorly soluble drugs (Sun et al., 2019). A summary of the types of nanoparticles susceptible to parasites is shown in **Table 2**.

**Table 2:** List of the types of nanoparticles susceptibility to protozoa parasites.

Type of nanoparticle	Drug	Parasite	Effect	Reference
**Polymeric nanoparticles**	Chitosan	*C. parvum*	Reduced the number of Cryptosporidium oocysts	(Ahmed et al., 2019)
**Polymeric nanoparticles**	Clofazimine	*C. parvum*	Increased solubility by 90 times	(Zhang et al., 2017; Feng et al., 2018)
**Nano-suspension**	Bupravaquone	*C. parvum*	Enhanced mucosal adsorption and targeting	(Lemke et al., 2010)
**Polymeric nanoparticles**	Nano-Nitazoxanide (NTZ)	*C. parvum*	decreased the number of parasites, been more effective at day 6 of treatment	(Sedighi et al., 2016)
**Polymeric nanoparticles**	antibody-engineered with Indinavir	*C. parvum*	were able to target C. parvum in infected cells	(Bondioli et al., 2011)
**Polymeric nanoparticles**	Silver	*C. parvum*	high concentrations are able to fully break the oocyst wall	(Cameron et al., 2016)
**Polymeric nanoparticles**	Cooper oxide and silver	*E. histolytica; C. parvum*	significant reduction for cysts viability	(Saad et al., 2015)
**Polymeric nanoparticles**	Silver, chitosan, and curcumin	*G. lamblia*	Highest effect was combining the three nanoform drugs. Parasite was eliminated from feces and intestine.	(Said et al., 2012)
**Polymeric nanoparticles**	Gold	*G. lamblia*	concentration of 0.3 mg/ml was effective to eliminated cysts	(Bavand et al., 2014)
**Polymeric nanoparticles**	Metronidazole	*G. lamblia*	completely eliminated cyst shedding and trophozoite count compared with Giardia-infected mice	(Madbouly et al., 2020)
**Nano-emulsion**	Micana cordifolia	*T. vaginalis*	anti-T. vaginalis activity was observed to be 100% at 1000 ppm	(Vazini, 2017)
**Polymeric nanoparticles**	Chitosan	*T. vaginalis*	showed a strong at 100 μg/mL	(Pradines et al., 2015)

## 7 Conclusion

Parasitic mucosa-associated protozoa cause severe health, social, and economic impacts, mainly in low-income settings that are resource constrained. Considering the scarcity drugs available to treat these diseases and the threat of resistant cases, the search for new therapeutics is urgently needed. However, despite considerable effort, no new novel drugs have been approved as a novel option for treatment. Also, there are no new compounds currently in clinical trials. Most of the targets and compounds were able to show some efficacy *in vitro*, *in vivo*, or both. Also, they have already proven effective in large animal models, although the compounds so far have not reached clinical application. Ahead, the huge challenge is to get the best candidates for clinical studies.

## Author Contributions

KMR and HLCS contributed equally to this work and approved the submitted version.

## Funding

This work was supported by the Fundação de Amparo à Pesquisa do Estado do Rio de Janeiro, Conselho Nacional de Desenvolvimento Científico e Tecnológico, Coordenação de Aperfeiçoamento de Pessoal de Nível Superior and Fundação Oswaldo Cruz.

## Conflict of Interest

The authors declare that the research was conducted in the absence of any commercial or financial relationships that could be construed as a potential conflict of interest.

## Publisher’s Note

All claims expressed in this article are solely those of the authors and do not necessarily represent those of their affiliated organizations, or those of the publisher, the editors and the reviewers. Any product that may be evaluated in this article, or claim that may be made by its manufacturer, is not guaranteed or endorsed by the publisher.

## Supplementary Material

Supplementary Table 1 List of natural compounds with antiprotozoa activity.

## References

[B1] Abid M. Agarwal S. M. Azam A. (2008). Synthesis and Antiamoebic Activity of Metronidazole Thiosemicarbazone Analogues. Eur. J. Med. Chem. 43, 2035–2039. doi: 10.1016/j.ejmech.2007.12.007 18242785

[B2] Abubakar I. Aliyu S. H. Arumugam C. Hunter P. R. Usman N. K. (2007). Prevention and Treatment of Cryptosporidiosis in Immunocompromised Patients. Cochrane Database Syst. Rev. 24 (1), CD004932. doi: 10.1002/14651858.CD004932.pub2 PMC12042072 17253532

[B3] Adagu I. S. Nolder D. Warhurst D. C. Rossignol J. F. (2002). *In Vitro* Activity of Nitazoxanide and Related Compounds Against Isolates of Giardia Intestinalis, Entamoeba Histolytica and Trichomonas Vaginalis. J. Antimicrob. Chemother. 49, 103–111. doi: 10.1093/jac/49.1.103 11751773

[B4] Agholi M. Hatam G. R. Motazedian M. H. (2013). Microsporidia and Coccidia as Causes of Persistence Diarrhea Among Liver Transplant Children: Incidence Rate and Species/Genotypes. Pediatr. Infect. Dis. J. 32 (2) 185–187. doi: 10.1097/INF.0b013e318273d95f 22982981

[B5] Ahmed S. A. El-Mahallawy H. S. Karanis P. (2019). Inhibitory Activity of Chitosan Nanoparticles Against Cryptosporidium Parvum Oocysts. Parasitol. Res. 118, 2053–2063. doi: 10.1007/s00436-019-06364-0 31187224

[B6] Alves M. S. D. Das Neves R. N. Sena-Lopes A. Domingues M. Casaril A. M. Segatto N. V.. (2020). Antiparasitic Activity of Furanyl N-Acylhydrazone Derivatives Against Trichomonas Vaginalis: *In Vitro* and in Silico Analyses. Parasitol. Vectors 13, 59. doi: 10.1186/s13071-020-3923-8 PMC7014680 32046788

[B7] Amadi B. Mwiya M. Sianongo S. Payne L. Watuka A. Katubulushi M.. (2009). High Dose Prolonged Treatment With Nitazoxanide is Not Effective for Cryptosporidiosis in HIV Positive Zambian Children: A Randomised Controlled Trial. BMC Infect. Dis. 9, 195. doi: 10.1186/1471-2334-9-195 19954529 PMC2794874

[B8] Andersson K. E. (1981). Pharmacokinetics of Nitroimidazoles. Spectrum of Adverse Reactions. Scand. J. Infect. Dis. Suppl. 26, 60–67. 6941457

[B9] Andrade R. M. Reed S. L. (2015). New Drug Target in Protozoan Parasites: The Role of Thioredoxin Reductase. Front. Microbiol. 6, 975. doi: 10.3389/fmicb.2015.00975 26483758 PMC4588103

[B10] Anthwal A. Rajesh U. C. Rawat M. S. Kushwaha B. Maikhuri J. P. Sharma V. L.. (2014). Novel Metronidazole-Chalcone Conjugates With Potential to Counter Drug Resistance in Trichomonas Vaginalis. Eur. J. Med. Chem. 79, 89–94. doi: 10.1016/j.ejmech.2014.03.076 24727243

[B11] Ashburn T. T. Thor K. B. (2004). Drug Repositioning: Identifying and Developing New Uses for Existing Drugs. Nat. Rev. Drug Discovery 3, 673–683. doi: 10.1038/nrd1468 15286734

[B12] Banik G. R. Stark D. Rashid H. Ellis J. T. (2014). Recent Advances in Molecular Biology of Parasitic Viruses. Infect. Disord. Drug Targets 14, 155–167. doi: 10.2174/1871526514666140713160905 25019235

[B13] Baragana B. Forte B. Choi R. Nakazawa Hewitt S. Bueren-Calabuig J. A. Pisco J. P.. (2019). Lysyl-tRNA Synthetase as a Drug Target in Malaria and Cryptosporidiosis. Proc. Natl. Acad. Sci. U.S.A. 116, 7015–7020. doi: 10.1073/pnas.1814685116 30894487 PMC6452685

[B14] Barrow P. Dujardin J. C. Fasel N. Greenwood A. D. Osterrieder K. Lomonossoff G.. (2020). Viruses of Protozoan Parasites and Viral Therapy: Is the Time Now Right? Virol. J. 17, 142. doi: 10.1186/s12985-020-01410-1 32993724 PMC7522927

[B15] Bavand Z. Gholami S. Honari S. Rahimi Esboei B. Torabi N. Borabadi H. (2014). Effect of Gold Nanoparticles on Giardia Lambliacyst stage in *In Vitro* ? Arak. Med. Univ J. 16, 27–37.

[B16] Benchimol M. Chang T. H. Alderete J. F. (2002). Trichomonas Vaginalis: Observation of Coexistence of Multiple Viruses in the Same Isolate. FEMS Microbiol. Lett. 215, 197–201. doi: 10.1111/j.1574-6968.2002.tb11391.x 12399035

[B17] Billet A. C. Salmon Rousseau A. Piroth L. Martins C. (2019). An Underestimated Sexually Transmitted Infection: Amoebiasis. BMJ Case Rep. 12 (5), e228942. doi: 10.1136/bcr-2018-228942 PMC6536245 31079045

[B18] Bird R. G. Mccaul T. F. Knight R. (1974). Proceedings: Rhabdo-Virus Like Particles of Entamoeba Histolytica. Trans. R. Soc. Trop. Med. Hyg. 68, 2. doi: 10.1016/0035-9203(74)90214-4 4362018

[B19] Bondioli L. Ludovisi A. Tosi G. Ruozi B. Forni F. Pozio E.. (2011). The Loading of Labelled Antibody-Engineered Nanoparticles With Indinavir Increases its *In Vitro* Efficacy Against Cryptosporidium Parvum. Parasitology 138, 1384–1391. doi: 10.1017/S0031182011001119 21819637

[B20] Bouma M. J. Snowdon D. Fairlamb A. H. Ackers J. P. (1998). Activity of Disulfiram (Bis(Diethylthiocarbamoyl)Disulphide) and Ditiocarb (Diethyldithiocarbamate) Against Metronidazole-Sensitive and -Resistant Trichomonas Vaginalis and Tritrichomonas Foetus. J. Antimicrob. Chemother. 42, 817–820. doi: 10.1093/jac/42.6.817 10052908

[B21] Buckner F. S. Ranade R. M. Gillespie J. R. Shibata S. Hulverson M. A. Zhang Z.. (2019). Optimization of Methionyl tRNA-Synthetase Inhibitors for Treatment of Cryptosporidium Infection. Antimicrob. Agents Chemother. 63 (4), e02061-18. doi: 10.1128/AAC.02061-18 30745384 PMC6437504

[B22] Cameron P. Gaiser B. K. Bhandari B. Bartley P. M. Katzer F. Bridle H. (2016). Silver Nanoparticles Decrease the Viability of Cryptosporidium Parvum Oocysts. Appl. Environ. Microbiol. 82, 431–437. doi: 10.1128/AEM.02806-15 26497464 PMC4711145

[B23] Cao L. Gong P. Li J. Zhang X. Zou X. Tuo W.. (2009). Giardia Canis: Ultrastructural Analysis of G. Canis Trophozoites Transfected With Full Length G. Canis Virus cDNA Transcripts. Exp. Parasitol. 123, 212–217. doi: 10.1016/j.exppara.2009.07.001 19619539

[B24] Capparelli E. V. Bricker-Ford R. Rogers M. J. Mckerrow J. H. Reed S. L. (2017). Phase I Clinical Trial Results of Auranofin, a Novel Antiparasitic Agent. Antimicrob. Agents Chemother. 61 (1), e01947-16. doi: 10.1128/AAC.01947-16 27821451 PMC5192119

[B25] Carter E. R. Nabarro L. E. Hedley L. Chiodini P. L. (2018). Nitroimidazole-Refractory Giardiasis: A Growing Problem Requiring Rational Solutions. Clin. Microbiol. Infect. 24, 37–42. doi: 10.1016/j.cmi.2017.05.028 28624613

[B26] Castillo-Villanueva A. Rufino-Gonzalez Y. Mendez S. T. Torres-Arroyo A. Ponce-Macotela M. Martinez-Gordillo M. N.. (2017). Disulfiram as a Novel Inactivator of Giardia Lamblia Triosephosphate Isomerase With Antigiardial Potential. Int. J. Parasitol. Drugs Drug Resist. 7, 425–432. doi: 10.1016/j.ijpddr.2017.11.003 29197728 PMC5727346

[B27] Cedillo-Rivera R. Chavez B. Gonzalez-Robles A. Tapia A. Yepez-Mulia L. (2002). *In Vitro* Effect of Nitazoxanide Against Entamoeba Histolytica, Giardia Intestinalis and Trichomonas Vaginalis Trophozoites. J. Eukaryot. Microbiol. 49, 201–208. doi: 10.1111/j.1550-7408.2002.tb00523.x 12120985

[B28] Choi R. Hulverson M. A. Huang W. Vidadala R. S. R. Whitman G. R. Barrett L. K.. (2020). Bumped Kinase Inhibitors as Therapy for Apicomplexan Parasitic Diseases: Lessons Learned. Int. J. Parasitol. 50, 413–422. doi: 10.1016/j.ijpara.2020.01.006 32224121 PMC8565993

[B29] Coelho D. D. (1997). Metronidazole Resistant Trichomoniasis Successfully Treated With Paromomycin. Genitourin Med. 73, 397–398. doi: 10.1136/sti.73.5.397 9534753 PMC1195902

[B30] Collaborators, G. B. D. D. D. (2017). Estimates of Global, Regional, and National Morbidity, Mortality, and Aetiologies of Diarrhoeal Diseases: A Systematic Analysis for the Global Burden of Disease Study 2015. Lancet Infect. Dis. 17, 909–948. doi: 10.1016/S1473-3099(17)30276-1 28579426 PMC5589208

[B31] Collier S. A. Deng L. Adam E. A. Benedict K. M. Beshearse E. M. Blackstock A. J.. (2021). Estimate of Burden and Direct Healthcare Cost of Infectious Waterborne Disease in the United States. Emerg. Infect. Dis. 27, 140–149. doi: 10.3201/eid2701.190676 33350905 PMC7774540

[B32] Conners E. E. Miller A. D. Balachandran N. Robinson B. M. Benedict K. M. (2021). Giardiasis Outbreaks - United State-2017. MMWR Morb. Mortal Wkly Rep. 70, 304–307. doi: 10.15585/mmwr.mm7009a2 33661866 PMC7948938

[B33] Croft S. L. Seifert K. Duchene M. (2003). Antiprotozoal Activities of Phospholipid Analogues. Mol. Biochem. Parasitol. 126, 165–172. doi: 10.1016/S0166-6851(02)00283-9 12615315

[B34] Das Neves R. N. Sena-Lopes A. Alves M. S. D. Da Rocha Fonseca B. Da Silva C. C. Casaril A. M.. (2020). 2’-Hydroxychalcones as an Alternative Treatment for Trichomoniasis in Association With Metronidazole. Parasitol. Res. 119, 725–736. doi: 10.1007/s00436-019-06568-4 31853622

[B35] Debnath A. Ndao M. Reed S. L. (2013). Reprofiled Drug Targets Ancient Protozoans: Drug Discovery for Parasitic Diarrheal Diseases. Gut. Microbes 4, 66–71. doi: 10.4161/gmic.22596 23137963 PMC3555889

[B36] Debnath A. Parsonage D. Andrade R. M. He C. Cobo E. R. Hirata K.. (2012). A High-Throughput Drug Screen for Entamoeba Histolytica Identifies a New Lead and Target. Nat. Med. 18, 956–960. doi: 10.1038/nm.2758 22610278 PMC3411919

[B37] Diawara E. H. Francois A. Stachulski A. V. Razakandrainibe R. Costa D. Favennec L.. (2021). Systemic Efficacy on Cryptosporidium Parvum Infection of Aminoxanide (RM-5061), a New Amino-Acid Ester Thiazolide Prodrug of Tizoxanide. Parasitology 148, 975–984. doi: 10.1017/S0031182021000524 33775260 PMC11010128

[B38] Dimasi J. A. Hansen R. W. Grabowski H. G. (2003). The Price of Innovation: New Estimates of Drug Development Costs. J. Health Econ. 22, 151–185. doi: 10.1016/S0167-6296(02)00126-1 12606142

[B39] Diptyanusa A. Sari I. P. (2021). Treatment of Human Intestinal Cryptosporidiosis: A Review of Published Clinical Trials. Int. J. Parasitol. Drugs Drug Resist. 17, 128–138. doi: 10.1016/j.ijpddr.2021.09.001 34562754 PMC8473663

[B40] Duque-Montano B. E. Gomez-Caro L. C. Sanchez-Sanchez M. Monge A. Hernandez-Baltazar E. Rivera G.. (2013). Synthesis and *In Vitro* Evaluation of New Ethyl and Methyl Quinoxaline-7-Carboxylate 1,4-Di-N-Oxide Against Entamoeba Histolytica. Bioorg. Med. Chem. 21, 4550–4558. doi: 10.1016/j.bmc.2013.05.036 23787289

[B41] Efstratiou A. Ongerth J. E. Karanis P. (2017). Waterborne Transmission of Protozoan Parasites: Review of Worldwide Outbreaks - An Update 2011-2016. Water Res. 114, 14–22. doi: 10.1016/j.watres.2017.01.036 28214721

[B42] Ehrenkaufer G. Li P. Stebbins E. E. Kangussu-Marcolino M. M. Debnath A. White C. V.. (2020). Identification of Anisomycin, Prodigiosin and Obatoclax as Compounds With Broad-Spectrum Anti-Parasitic Activity. PloS Negl. Trop. Dis. 14, e0008150. doi: 10.1371/journal.pntd.0008150 32196500 PMC7112225

[B43] Ehrenkaufer G. M. Suresh S. Solow-Cordero D. Singh U. (2018). High-Throughput Screening of Entamoeba Identifies Compounds Which Target Both Life Cycle Stages and Which Are Effective Against Metronidazole Resistant Parasites. Front. Cell Infect. Microbiol. 8, 276. doi: 10.3389/fcimb.2018.00276 30175074 PMC6107840

[B44] El-Gayar E. K. Mokhtar A. B. Hassan W. A. (2016). Molecular Characterization of Double-Stranded RNA Virus in Trichomonas Vaginalis Egyptian Isolates and its Association With Pathogenicity. Parasitol. Res. 115, 4027–4036. doi: 10.1007/s00436-016-5174-3 27316695

[B45] Escobedo A. A. Almirall P. Alfonso M. Cimerman S. Chacin-Bonilla L. (2014). Sexual Transmission of Giardiasis: A Neglected Route of Spread? Acta Trop. 132, 106–111. doi: 10.1016/j.actatropica.2013.12.025 24434784

[B46] Escobedo A. A. Lalle M. Hrastnik N. I. Rodriguez-Morales A. J. Castro-Sanchez E. Cimerman S.. (2016). Combination Therapy in the Management of Giardiasis: What Laboratory and Clinical Studies Tell Us, So Far. Acta Trop. 162, 196–205. doi: 10.1016/j.actatropica.2016.06.026 27349189

[B47] Fantinatti M. Lopes-Oliveira L. A. P. Cascais-Figueredo T. Austriaco-Teixeira P. Verissimo E. Bello A. R.. (2020). Recirculation of Giardia Lamblia Assemblage A After Metronidazole Treatment in an Area With Assemblages A, B, and E Sympatric Circulation. Front. Microbiol. 11, 571104. doi: 10.3389/fmicb.2020.571104 33193167 PMC7642054

[B48] Feng J. Zhang Y. Mcmanus S. A. Ristroph K. D. Lu H. D. Gong K.. (2018). Rapid Recovery of Clofazimine-Loaded Nanoparticles With Long-Term Storage Stability as Anti-Cryptosporidium Therapy. ACS Appl. Nano Mater. 1, 2184–2194. doi: 10.1021/acsanm.8b00234 29911689 PMC5999231

[B49] Fichorova R. Fraga J. Rappelli P. Fiori P. L. (2017). Trichomonas Vaginalis Infection in Symbiosis With Trichomonasvirus and Mycoplasma. Res. Microbiol. 168, 882–891. doi: 10.1016/j.resmic.2017.03.005 28366838 PMC8130574

[B50] Fichorova R. N. Lee Y. Yamamoto H. S. Takagi Y. Hayes G. R. Goodman R. P.. (2012). Endobiont Viruses Sensed by the Human Host - Beyond Conventional Antiparasitic Therapy. PloS One 7, e48418. doi: 10.1371/journal.pone.0048418 23144878 PMC3492353

[B51] Fraga J. Rojas L. Sariego I. Fernandez-Calienes A. Nunez F. A. (2012). Species Typing of Cuban Trichomonas Vaginalis Virus by RT-PCR, and Association of TVV-2 With High Parasite Adhesion Levels and High Pathogenicity in Patients. Arch. Virol. 157, 1789–1795. doi: 10.1007/s00705-012-1353-4 22653538

[B52] Funkhouser-Jones L. J. Ravindran S. Sibley L. D. (2020). Defining Stage-Specific Activity of Potent New Inhibitors of Cryptosporidium Parvum Growth *In Vitro* . mBio 11 (2), e00052-20. doi: 10.1128/mBio.00052-20 32127445 PMC7064746

[B53] Gargala G. Le Goff L. Ballet J. J. Favennec L. Stachulski A. V. Rossignol J. F. (2010). Evaluation of New Thiazolide/Thiadiazolide Derivatives Reveals Nitro Group-Independent Efficacy Against *In Vitro* Development of Cryptosporidium Parvum. Antimicrob. Agents Chemother. 54, 1315–1318. doi: 10.1128/AAC.00614-09 20047919 PMC2825981

[B54] Gavrilov O. K. (1989). Gravitational Surgical Correction of the Aggregative Status of the Blood in Ischemic Heart Disease. Klin. Med. (Mosk) 67, 8–12. 2615315

[B55] Gharpure R. Perez A. Miller A. D. Wikswo M. E. Silver R. Hlavsa M. C. (2019). Cryptosporidiosis Outbreaks - United States 2009-2017. MMWR Morb. Mortal Wkly Rep. 68, 568–572. doi: 10.15585/mmwr.mm6825a3 31246941 PMC6597118

[B56] Ghosh S. Farr L. Singh A. Leaton L. A. Padalia J. Shirley D. A.. (2020). COP9 Signalosome is an Essential and Druggable Parasite Target That Regulates Protein Degradation. PloS Pathog. 16, e1008952. doi: 10.1371/journal.ppat.1008952 32960936 PMC7531848

[B57] Gomez-Arreaza A. Haenni A. L. Dunia I. Avilan L. (2017). Viruses of Parasites as Actors in the Parasite-Host Relationship: A "Menage a Trois". Acta Trop. 166, 126–132. doi: 10.1016/j.actatropica.2016.11.028 27876650

[B58] Gonzales M. L. M. Dans L. F. Sio-Aguilar J. (2019). Antiamoebic Drugs for Treating Amoebic Colitis. Cochrane Database Syst. Rev. 1, CD006085. doi: 10.1002/14651858.CD006085.pub3 30624763 PMC6326239

[B59] Gonzalez Constandse R. (1956). Anisomycin in Intestinal Amebiasis; Study of 30 Clinical Cases. Prensa Med. Mex 21, 114–115. 13408199

[B60] Goodgame R. (2003). Emerging Causes of Traveler’s Diarrhea: Cryptosporidium, Cyclospora, Isospora, and Microsporidia. Curr. Infect. Dis. Rep. 5, 66–73. doi: 10.1007/s11908-003-0067-x 12525293

[B61] Govender Y. Chan T. Yamamoto H. S. Budnik B. Fichorova R. N. (2020). The Role of Small Extracellular Vesicles in Viral-Protozoan Symbiosis: Lessons From Trichomonasvirus in an Isogenic Host Parasite Model. Front. Cell Infect. Microbiol. 10, 591172. doi: 10.3389/fcimb.2020.591172 33224901 PMC7674494

[B62] Graves K. J. Ghosh A. P. Schmidt N. Augostini P. Secor W. E. Schwebke J. R.. (2019). Trichomonas Vaginalis Virus Among Women With Trichomoniasis and Associations With Demographics, Clinical Outcomes, and Metronidazole Resistance. Clin. Infect. Dis. 69, 2170–2176. doi: 10.1093/cid/ciz146 30768180 PMC6880324

[B63] Gulmezoglu A. M. Garner P. (1998). Trichomoniasis Treatment in Women: A Systematic Review. Trop. Med. Int. Health 3, 553–558. doi: 10.1046/j.1365-3156.1998.00273.x 9705189

[B64] Hahn J. Seeber F. Kolodziej H. Ignatius R. Laue M. Aebischer T.. (2013). High Sensitivity of Giardia Duodenalis to Tetrahydrolipstatin (Orlistat) *In Vitro* . PloS One 8, e71597. doi: 10.1371/journal.pone.0071597 23977083 PMC3747212

[B65] Haque R. Huston C. D. Hughes M. Houpt E. Petri W. A. Jr. (2003). Amebiasis. N. Engl. J. Med. 348, 1565–1573. doi: 10.1056/NEJMra022710 12700377

[B66] Hernandez Ceruelos A. Romero-Quezada L. C. Ruvalcaba Ledezma J. C. Lopez Contreras L. (2019). Therapeutic Uses of Metronidazole and its Side Effects: An Update. Eur. Rev. Med. Pharmacol. Sci. 23, 397–401. doi: 10.26355/eurrev_201901_16788 30657582

[B67] Hernandez-Ochoa B. Gomez-Manzo S. Sanchez-Carrillo A. Marcial-Quino J. Rocha-Ramirez L. M. Santos-Segura A.. (2020). Enhanced Antigiardial Effect of Omeprazole Analog Benzimidazole Compounds. Molecules 25 (17), 3979. doi: 10.3390/molecules25173979 PMC7504735 32882836

[B68] Hernandez-Ochoa B. Navarrete-Vazquez G. Nava-Zuazo C. Castillo-Villanueva A. Mendez S. T. Torres-Arroyo A.. (2017). Novel Giardicidal Compounds Bearing Proton Pump Inhibitor Scaffold Proceeding Through Triosephosphate Isomerase Inactivation. Sci. Rep. 7, 7810. doi: 10.1038/s41598-017-07612-y 28798383 PMC5552691

[B69] Hlavsa M. C. Roellig D. M. Seabolt M. H. Kahler A. M. Murphy J. L. Mckitt T. K.. (2017). Using Molecular Characterization to Support Investigations of Aquatic Facility-Associated Outbreaks of Cryptosporidiosis - Alabama, Arizona, and Ohi. MMWR Morb. Mortal Wkly Rep. 66, 493–497. doi: 10.15585/mmwr.mm6619a2 28520707 PMC5657643

[B70] Horner D. S. Hirt R. P. Embley T. M. (1999). A Single Eubacterial Origin of Eukaryotic Pyruvate: Ferredoxin Oxidoreductase Genes: Implications for the Evolution of Anaerobic Eukaryotes. Mol. Biol. Evol. 16, 1280–1291. doi: 10.1093/oxfordjournals.molbev.a026218 10486982

[B71] Hung C. C. Chang S. Y. Ji D. D. (2012). Entamoeba Histolytica Infection in Men Who Have Sex With Men. Lancet Infect. Dis. 12, 729–736. doi: 10.1016/S1473-3099(12)70147-0 22917103

[B72] Huston C. D. (2021). The Clofazimine for Treatment of Cryptosporidiosis in HIV-Infected Adults (CRYPTOFAZ) and Lessons Learned for Anticryptosporidial Drug Development. Clin. Infect. Dis. 73, 192–194. doi: 10.1093/cid/ciaa425 32277815 PMC8427724

[B73] Innes E. A. Chalmers R. M. Wells B. Pawlowic M. C. (2020). A One Health Approach to Tackle Cryptosporidiosis. Trends Parasitol. 36, 290–303. doi: 10.1016/j.pt.2019.12.016 31983609 PMC7106497

[B74] Iyer LR Banyal N. Naik S. J. P. (2017). Antioxidant Enzyme Profile of Two Clinical Isolates of Entamoeba Histolytica Varying in Sensitivity to Antiamoebic Drugs. World J. Clin. Infect. Dis. 7, 6. doi: 10.5495/wjcid.v7.i2.21

[B75] Jain V. Yogavel M. Kikuchi H. Oshima Y. Hariguchi N. Matsumoto M.. (2017). Targeting Prolyl-tRNA Synthetase to Accelerate Drug Discovery Against Malaria, Leishmaniasis, Toxoplasmosis, Cryptosporidiosis, and Coccidiosis. Structure 25, 1495–1505 e6. doi: 10.1016/j.str.2017.07.015 28867614

[B76] Jefferies R. Yang R. Woh C. K. Weldt T. Milech N. Estcourt A.. (2015). Target Validation of the Inosine Monophosphate Dehydrogenase (IMPDH) Gene in Cryptosporidium Using Phylomer((R)) Peptides. Exp. Parasitol. 148, 40–48. doi: 10.1016/j.exppara.2014.11.003 25447124

[B77] Jenkins M. C. O’brien C. N. Santin M. Fayer R. (2015). Changes in the Levels of Cryspovirus During *In Vitro* Development of Cryptosporidium Parvum. Parasitol. Res. 114, 2063–2068. doi: 10.1007/s00436-015-4390-6 25704645

[B78] Jimenez-Cardoso E. Eligio-Garcia L. Cortes-Campos A. Flores-Luna A. Valencia-Mayoral P. Lozada-Chavez I. (2009). Changes in Beta-Giardin Sequence of Giardia Intestinalis Sensitive and Resistant to Albendazole Strains. Parasitol. Res. 105, 25–33. doi: 10.1007/s00436-009-1363-7 19214572

[B79] Jourdan J. P. Bureau R. Rochais C. Dallemagne P. (2020). Drug Repositioning: A Brief Overview. J. Pharm. Pharmacol. 72, 1145–1151. doi: 10.1111/jphp.13273 32301512 PMC7262062

[B80] Jumani R. S. Bessoff K. Love M. S. Miller P. Stebbins E. E. Teixeira J. E.. (2018). A Novel Piperazine-Based Drug Lead for Cryptosporidiosis From the Medicines for Malaria Venture Open-Access Malaria Box. Antimicrob. Agents Chemother. 62 (4), e01505-17. doi: 10.1128/AAC.01505-17 29339392 PMC5913971

[B81] Kappagoda S. Singh U. Blackburn B. G. (2011). Antiparasitic Therapy. Mayo Clin. Proc. 86, 561–583. doi: 10.4065/mcp.2011.0203 21628620 PMC3104918

[B82] Karanis P. Kourenti C. Smith H. (2007). Waterborne Transmission of Protozoan Parasites: A Worldwide Review of Outbreaks and Lessons Learnt. J. Water Health 5, 1–38. doi: 10.2166/wh.2006.002 17402277

[B83] Khare S. Nagle A. S. Biggart A. Lai Y. H. Liang F. Davis L. C.. (2016). Proteasome Inhibition for Treatment of Leishmaniasis, Chagas Disease and Sleeping Sickness. Nature 537, 229–233. doi: 10.1038/nature19339 27501246 PMC5161665

[B84] Kirkcaldy R. D. Augostini P. Asbel L. E. Bernstein K. T. Kerani R. P. Mettenbrink C. J.. (2012). Trichomonas Vaginalis Antimicrobial Drug Resistance in 6 US Cities, STD Surveillance Network 2009-2010. Emerg. Infect. Dis. 18, 939–943. doi: 10.3201/eid1806.111590 22608054 PMC3358158

[B85] Kissinger P. Adamski A. (2013). Trichomoniasis and HIV Interactions: A Review. Sex Transm. Infect. 89, 426–433. doi: 10.1136/sextrans-2012-051005 23605851 PMC3748151

[B86] Kuhlenschmidt T. B. Rutaganira F. U. Long S. Tang K. Shokat K. M. Kuhlenschmidt M. S.. (2016). Inhibition of Calcium-Dependent Protein Kinase 1 (CDPK1) *In Vitro* by Pyrazolopyrimidine Derivatives Does Not Correlate With Sensitivity of Cryptosporidium Parvum Growth in Cell Culture. Antimicrob. Agents Chemother. 60, 570–579. doi: 10.1128/AAC.01915-15 26552986 PMC4704151

[B87] Kulakova L. Galkin A. Chen C. Z. Southall N. Marugan J. J. Zheng W.. (2014). Discovery of Novel Antigiardiasis Drug Candidates. Antimicrob. Agents Chemother. 58, 7303–7311. doi: 10.1128/AAC.03834-14 25267663 PMC4249522

[B88] Kumar S. Bains T. Won Kim A. S. Tam C. Kim J. Cheng L. W.. (2018). Highly Potent 1h-1,2,3-Triazole-Tethered Isatin-Metronidazole Conjugates Against Anaerobic Foodborne, Waterborne, and Sexually-Transmitted Protozoal Parasites. Front. Cell Infect. Microbiol. 8, 380. doi: 10.3389/fcimb.2018.00380 30425970 PMC6218680

[B89] Lalle M. Hanevik K. (2018). Treatment-Refractory Giardiasis: Challenges and Solutions. Infect. Drug Resist. 11, 1921–1933. doi: 10.2147/IDR.S141468 30498364 PMC6207226

[B90] Lazenby G. B. Thompson L. Powell A. M. Soper D. E. (2019). Unexpected High Rates of Persistent Trichomonas Vaginalis Infection in a Retrospective Cohort of Treated Pregnant Women. Sex Transm. Dis. 46, 2–8. doi: 10.1097/OLQ.0000000000000902 30067546

[B91] Leitsch D. (2015). Drug Resistance in the Microaerophilic Parasite Giardia Lamblia. Curr. Trop. Med. Rep. 2, 128–135. doi: 10.1007/s40475-015-0051-1 26258002 PMC4523694

[B92] Leitsch D. (2016). Recent Advances in the Trichomonas Vaginalis Field. F1000Res 5 (F1000 Faculty Rev), 162. doi: 10.12688/f1000research.7594.1 PMC4755396 26918168

[B93] Leitsch D. (2019). A Review on Metronidazole: An Old Warhorse in Antimicrobial Chemotherapy. Parasitology 146, 1167–1178. doi: 10.1017/S0031182017002025 29166971

[B94] Leitsch D. Burgess A. G. Dunn L. A. Krauer K. G. Tan K. Duchene M.. (2011). Pyruvate:ferredoxin Oxidoreductase and Thioredoxin Reductase are Involved in 5-Nitroimidazole Activation While Flavin Metabolism is Linked to 5-Nitroimidazole Resistance in Giardia Lamblia. J. Antimicrob. Chemother. 66, 1756–1765. doi: 10.1093/jac/dkr192 21602576 PMC3133484

[B95] Leitsch D. Drinic M. Kolarich D. Duchene M. (2012). Down-Regulation of Flavin Reductase and Alcohol Dehydrogenase-1 (ADH1) in Metronidazole-Resistant Isolates of Trichomonas Vaginalis. Mol. Biochem. Parasitol. 183, 177–183. doi: 10.1016/j.molbiopara.2012.03.003 22449940 PMC3341570

[B96] Leitsch D. Janssen B. D. Kolarich D. Johnson P. J. Duchene M. (2014). Trichomonas Vaginalis Flavin Reductase 1 and its Role in Metronidazole Resistance. Mol. Microbiol. 91, 198–208. doi: 10.1111/mmi.12455 24256032 PMC4437529

[B97] Lemee V. Zaharia I. Nevez G. Rabodonirina M. Brasseur P. Ballet J. J.. (2000). Metronidazole and Albendazole Susceptibility of 11 Clinical Isolates of Giardia Duodenalis From France. J. Antimicrob. Chemother. 46, 819–821. doi: 10.1093/jac/46.5.819 11062206

[B98] Lemke A. Kiderlen A. F. Petri B. Kayser O. (2010). Delivery of Amphotericin B Nanosuspensions to the Brain and Determination of Activity Against Balamuthia Mandrillaris Amebas. Nanomedicine 6, 597–603. doi: 10.1016/j.nano.2009.12.004 20060497

[B99] Leung A. K. C. Leung A. A. M. Wong A. H. C. Sergi C. M. Kam J. K. M. (2019). Giardiasis: An Overview. Recent Pat. Inflammation Allergy Drug Discovery 13, 134–143. doi: 10.2174/1872213X13666190618124901 31210116

[B100] Li J. Cui Z. Li X. Zhang L. (2021). Review of Zoonotic Amebiasis: Epidemiology, Clinical Signs, Diagnosis, Treatment, Prevention and Control. Res. Vet. Sci. 136, 174–181. doi: 10.1016/j.rvsc.2021.02.021 33676155

[B101] Lin H. C. Chu L. J. Huang P. J. Cheng W. H. Zheng Y. H. Huang C. Y.. (2020). Proteomic Signatures of Metronidazole-Resistant Trichomonas Vaginalis Reveal Novel Proteins Associated With Drug Resistance. Parasitol. Vectors 13, 274. doi: 10.1186/s13071-020-04148-5 PMC7268490 32487244

[B102] Liu H. W. Chu Y. D. Tai J. H. (1998). Characterization of Trichomonas Vaginalis Virus Proteins in the Pathogenic Protozoan T. Vaginalis. Arch. Virol. 143, 963–970. doi: 10.1007/s007050050345 9645201

[B103] Liu J. Kanetake S. Wu Y. H. Tam C. Cheng L. W. Land K. M.. (2016). Antiprotozoal Effects of the Tomato Tetrasaccharide Glycoalkaloid Tomatine and the Aglycone Tomatidine on Mucosal Trichomonads. J. Agric. Food Chem. 64, 8806–8810. doi: 10.1021/acs.jafc.6b04030 27934291

[B104] Lofmark S. Edlund C. Nord C. E. (2010). Metronidazole is Still the Drug of Choice for Treatment of Anaerobic Infections. Clin. Infect. Dis. 50 Suppl 1, S16–S23. doi: 10.1086/647939 20067388

[B105] Lopez-Velazquez G. Fernandez-Lainez C. de la Mora-De La Mora J. I. Caudillo de la Portilla D. Reynoso-Robles R. Gonzalez-Maciel A.. (2019). On the Molecular and Cellular Effects of Omeprazole to Further Support its Effectiveness as an Antigiardial Drug. Sci. Rep. 9, 8922. doi: 10.1038/s41598-019-45529-w 31222100 PMC6586891

[B106] Love M. S. Choy R. K. M. (2021). Emerging Treatment Options for Cryptosporidiosis. Curr. Opin. Infect. Dis. 34, 455–462. doi: 10.1097/QCO.0000000000000761 34261904 PMC7611666

[B107] Ludvik J. Shipstone A. C. (1970). The Ultrastructure of Entamoeba Histolytica. Bull. World Health Organ 43, 301–308. 4320960 PMC2427647

[B108] Mac Kenzie W. R. Hoxie N. J. Proctor M. E. Gradus M. S. Blair K. A. Peterson D. E.. (1994). A Massive Outbreak in Milwaukee of Cryptosporidium Infection Transmitted Through the Public Water Supply. N. Engl. J. Med. 331, 161–167. doi: 10.1056/NEJM199407213310304 7818640

[B109] Madbouly N. A. Nashee H. Elgendy A. A. Rabee I. El Amir A. (2020). Encapsulation of Low Metronidazole Dose in Poly (D,L-Lactide-Co-Glycolide) (PLGA) Nanoparticles Improves Giardia Intestinalis Treatment. Infect. Chemother. 52, 550–561. doi: 10.3947/ic.2020.52.4.550 33377322 PMC7779986

[B110] Malla N. Kaul P. Sehgal R. Gupta I. (2011). The Presence of dsRNA Virus in Trichomonas Vaginalis Isolates From Symptomatic and Asymptomatic Indian Women and its Correlation With *In Vitro* Metronidazole Sensitivity. Indian J. Med. Microbiol. 29, 152–157. doi: 10.4103/0255-0857.81801 21654110

[B111] Manjunatha U. H. Vinayak S. Zambriski J. A. Chao A. T. Sy T. Noble C. G.. (2017). A Cryptosporidium Pi(4)K Inhibitor is a Drug Candidate for Cryptosporidiosis. Nature 546, 376–380. doi: 10.1038/nature22337 28562588 PMC5473467

[B112] Martinez-Perez Y. Nequiz-Avendano M. Garcia-Torres I. Gudino-Zayas M. E. Lopez-Velazquez G. Enriquez-Flores S.. (2020). Rabeprazole Inhibits Several Functions of Entamoeba Histolytica Related With its Virulence. Parasitol. Res. 119, 3491–3502. doi: 10.1007/s00436-020-06868-0 32886229

[B113] Marucci G. Zullino I. Bertuccini L. Camerini S. Cecchetti S. Pietrantoni A.. (2021). Re-Discovery of Giardiavirus: Genomic and Functional Analysis of Viruses From Giardia Duodenalis Isolates. Biomedicines 9 (6), 654. doi: 10.3390/biomedicines9060654 34201207 PMC8230311

[B114] Matadamas-Martinez F. Castillo R. Hernandez-Campos A. Mendez-Cuesta C. De Souza W. Gadelha A. P.. (2016). Proteomic and Ultrastructural Analysis of the Effect of a New Nitazoxanide-N-Methyl-1H-Benzimidazole Hybrid Against Giardia Intestinalis. Res. Vet. Sci. 105, 171–179. doi: 10.1016/j.rvsc.2016.02.006 27033928

[B115] Meites E. Gaydos C. A. Hobbs M. M. Kissinger P. Nyirjesy P. Schwebke J. R.. (2015). A Review of Evidence-Based Care of Symptomatic Trichomoniasis and Asymptomatic Trichomonas Vaginalis Infections. Clin. Infect. Dis. 61 (Suppl 8), S837–S848. doi: 10.1093/cid/civ738 26602621 PMC4657597

[B116] Meneses-Marcel A. Marrero-Ponce Y. Ibanez-Escribano A. Gomez-Barrio A. Escario J. A. Barigye S. J.. (2018). Drug Repositioning for Novel Antitrichomonas From Known Antiprotozoan Drugs Using Hierarchical Screening. Future Med. Chem. 10, 863–878. doi: 10.4155/fmc-2016-0211 29589477

[B117] Meri T. Jokiranta T. S. Suhonen L. Meri S. (2000). Resistance of Trichomonas Vaginalis to Metronidazole: Report of the First Three Cases From Finland and Optimization of *In Vitro* Susceptibility Testing Under Various Oxygen Concentrations. J. Clin. Microbiol. 38, 763–767. doi: 10.1128/JCM.38.2.763-767.2000 10655382 PMC86198

[B118] Mielczarek E. Blaszkowska J. (2016). Trichomonas Vaginalis: Pathogenicity and Potential Role in Human Reproductive Failure. Infection 44, 447–458. doi: 10.1007/s15010-015-0860-0 26546373

[B119] Morch K. Hanevik K. Robertson L. J. Strand E. A. Langeland N. (2008). Treatment-Ladder and Genetic Characterisation of Parasites in Refractory Giardiasis After an Outbreak in Norway. J. Infect. 56, 268–273. doi: 10.1016/j.jinf.2008.01.013 18328567

[B120] Morgan U. M. Reynoldson J. A. Thompson R. C. (1993). Activities of Several Benzimidazoles and Tubulin Inhibitors Against Giardia Spp. *In Vitro* . Antimicrob. Agents Chemother. 37, 328–331. doi: 10.1128/AAC.37.2.328 8452365 PMC187662

[B121] Mori M. Tsuge S. Fukasawa W. Jeelani G. Nakada-Tsukui K. Nonaka K.. (2018). Discovery of Antiamebic Compounds That Inhibit Cysteine Synthase From the Enteric Parasitic Protist Entamoeba Histolytica by Screening of Microbial Secondary Metabolites. Front. Cell Infect. Microbiol. 8, 409. doi: 10.3389/fcimb.2018.00409 30568921 PMC6290340

[B122] Muller J. Hemphill A. (2013). New Approaches for the Identification of Drug Targets in Protozoan Parasites. Int. Rev. Cell Mol. Biol. 301, 359–401. doi: 10.1016/B978-0-12-407704-1.00007-5 23317822

[B123] Muller J. Ley S. Felger I. Hemphill A. Muller N. (2008). Identification of Differentially Expressed Genes in a Giardia Lamblia WB C6 Clone Resistant to Nitazoxanide and Metronidazole. J. Antimicrob. Chemother. 62, 72–82. doi: 10.1093/jac/dkn142 18408240

[B124] Muller J. Ruhle G. Muller N. Rossignol J. F. Hemphill A. (2006). *In Vitro* Effects of Thiazolides on Giardia Lamblia WB Clone C6 Cultured Axenically and in Coculture With Caco2 Cells. Antimicrob. Agents Chemother. 50, 162–170. doi: 10.1128/AAC.50.1.162-170.2006 16377682 PMC1346829

[B125] Muller J. Wastling J. Sanderson S. Muller N. Hemphill A. (2007). A Novel Giardia Lamblia Nitroreductase, GlNR1, Interacts With Nitazoxanide and Other Thiazolides. Antimicrob. Agents Chemother. 51, 1979–1986. doi: 10.1128/AAC.01548-06 17438059 PMC1891416

[B126] Nabarro L. E. Lever R. A. Armstrong M. Chiodini P. L. (2015). Increased Incidence of Nitroimidazole-Refractory Giardiasis at the Hospital for Tropical Diseases, London: 2008-2013. Clin. Microbiol. Infect. 21, 791–796. doi: 10.1016/j.cmi.2015.04.019 25975511

[B127] Ndao M. Nath-Chowdhury M. Sajid M. Marcus V. Mashiyama S. T. Sakanari J.. (2013). A Cysteine Protease Inhibitor Rescues Mice From a Lethal Cryptosporidium Parvum Infection. Antimicrob. Agents Chemother. 57, 6063–6073. doi: 10.1128/AAC.00734-13 24060869 PMC3837922

[B128] Newman L. Rowley J. Vander Hoorn S. Wijesooriya N. S. Unemo M. Low N.. (2015). Global Estimates of the Prevalence and Incidence of Four Curable Sexually Transmitted Infections in 2012 Based on Systematic Review and Global Reporting. PloS One 10, e0143304. doi: 10.1371/journal.pone.0143304 26646541 PMC4672879

[B129] Nibert M. L. Woods K. M. Upton S. J. Ghabrial S. A. (2009). Cryspovirus: A New Genus of Protozoan Viruses in the Family Partitiviridae. Arch. Virol. 154, 1959–1965. doi: 10.1007/s00705-009-0513-7 19856142

[B130] Nillius D. Muller J. Muller N. (2011). Nitroreductase (GlNR1) Increases Susceptibility of Giardia Lamblia and Escherichia Coli to Nitro Drugs. J. Antimicrob. Chemother. 66, 1029–1035. doi: 10.1093/jac/dkr029 21393225

[B131] Nisha G. Mehra V. Hopper M. Patel N. Hall D. Wrischnik L. A.. (2013). Design and Synthesis of B-Amino Alcohol Based B-Lactam-Isatin Chimeras and Preliminary Analysis of *In Vitro* Activity Against the Protozoal Pathogen Trichomonas Vaginalis. Med. Chem. Commun. 4, 1018–1024. doi: 10.1039/c3md00057e

[B132] O’donoghue A. J. Bibo-Verdugo B. Miyamoto Y. Wang S. C. Yang J. Z. Zuill D. E.. (2019). 20s Proteasome as a Drug Target in Trichomonas Vaginalis. Antimicrob. Agents Chemother. 63 (11), e00448-19. doi: 10.1128/AAC.00448-19 31451503 PMC6811414

[B133] Oliveira Y. Oliveira L. M. Oliveira Y. L. M. Nascimento A. M. D. La Corte R. Geraldi R. M.. (2020). Changes in the Epidemiological Profile of Intestinal Parasites After a School-Based Large-Scale Treatment for Soil-Transmitted Helminths in a Community in Northeastern Brazil: Epidemiological Profile After Large-Scale School-Based Treatment for STH. Acta Trop. 202, 105279. doi: 10.1016/j.actatropica.2019.105279 31758913

[B134] Painter J. E. Gargano J. W. Yoder J. S. Collier S. A. Hlavsa M. C. (2016). Evolving Epidemiology of Reported Cryptosporidiosis Cases in the United State-2012. Epidemiol. Infect. 144, 1792–1802. doi: 10.1017/S0950268815003131 27125575 PMC9150710

[B135] Painter J. E. Hlavsa M. C. Collier S. A. Xiao L. Yoder J. S. Centers For Disease, C. & Prevention (2015). Cryptosporidiosis Surveillance – United States 2011-2012. MMWR Suppl. 64, 1–14. 25928581

[B136] Palencia A. Liu R. J. Lukarska M. Gut J. Bougdour A. Touquet B.. (2016). Cryptosporidium and Toxoplasma Parasites Are Inhibited by a Benzoxaborole Targeting Leucyl-tRNA Synthetase. Antimicrob. Agents Chemother. 60, 5817–5827. doi: 10.1128/AAC.00873-16 27431220 PMC5038320

[B137] Paulish-Miller T. E. Augostini P. Schuyler J. A. Smith W. L. Mordechai E. Adelson M. E.. (2014). Trichomonas Vaginalis Metronidazole Resistance is Associated With Single Nucleotide Polymorphisms in the Nitroreductase Genes Ntr4tv and Ntr6tv. Antimicrob. Agents Chemother. 58, 2938–2943. doi: 10.1128/AAC.02370-13 24550324 PMC3993245

[B138] Pawlowic M. C. Somepalli M. Sateriale A. Herbert G. T. Gibson A. R. Cuny G. D.. (2019). Genetic Ablation of Purine Salvage in Cryptosporidium Parvum Reveals Nucleotide Uptake From the Host Cell. Proc. Natl. Acad. Sci. U.S.A. 116, 21160–21165. doi: 10.1073/pnas.1908239116 31570573 PMC6800313

[B139] Perez S. Fernandez-Verdugo A. Perez F. Vazquez F. (2001). Prevalence of 5-Nitroimidazole-Resistant Trichomonas Vaginalis in Oviedo, Spain. Sex Transm. Dis. 28, 115–116. doi: 10.1097/00007435-200102000-00010 11234785

[B140] Perez-Villanueva J. Yepez-Mulia L. Gonzalez-Sanchez I. Palacios-Espinosa J. F. Soria-Arteche O. Sainz-Espunes T. D. R.. (2017). Synthesis and Biological Evaluation of 2H-Indazole Derivatives: Towards Antimicrobial and Anti-Inflammatory Dual Agents. Molecules 22 (11), 1864. doi: 10.3390/molecules22111864 PMC6150295 29088121

[B141] Pradines B. Bories C. Vauthier C. Ponchel G. Loiseau P. M. Bouchemal K. (2015). Drug-Free Chitosan Coated Poly(Isobutylcyanoacrylate) Nanoparticles are Active Against Trichomonas Vaginalis and non-Toxic Towards Pig Vaginal Mucosa. Pharm. Res. 32, 1229–1236. doi: 10.1007/s11095-014-1528-7 25319099

[B142] Provenzano D. Khoshnan A. Alderete J. F. (1997). Involvement of dsRNA Virus in the Protein Composition and Growth Kinetics of Host Trichomonas Vaginalis. Arch. Virol. 142, 939–952. doi: 10.1007/s007050050130 9191859

[B143] Quihui-Cota L. Morales-Figueroa G. G. (2012). Persistence of Intestinal Parasitic Infections During the National De-Worming Campaign in Schoolchildren of Northwestern Mexico: A Cross-Sectional Study. Ann. Gastroenterol. 25, 57–60. 24714136 PMC3959347

[B144] Rasoloson D. Vanacova S. Tomkova E. Razga J. Hrdy I. Tachezy J.. (2002). Mechanisms of *In Vitro* Development of Resistance to Metronidazole in Trichomonas Vaginalis. Microbiol. (Reading) 148, 2467–2477. doi: 10.1099/00221287-148-8-2467 12177340

[B145] Reyes-Vivas H. de la Mora-De La Mora I. Castillo-Villanueva A. Yepez-Mulia L. Hernandez-Alcantara G. Figueroa-Salazar R.. (2014). Giardial Triosephosphate Isomerase as Possible Target of the Cytotoxic Effect of Omeprazole in Giardia Lamblia. Antimicrob. Agents Chemother. 58, 7072–7082. doi: 10.1128/AAC.02900-14 25223993 PMC4249547

[B146] Rodriguez-Villar K. Yepez-Mulia L. Cortes-Gines M. Aguilera-Perdomo J. D. Quintana-Salazar E. A. Olascoaga Del Angel K. S.. (2021). Synthesis, Antiprotozoal Activity, and Cheminformatic Analysis of 2-Phenyl-2h-Indazole Derivatives. Molecules 26 (8), 2145. doi: 10.3390/molecules26082145 33917871 PMC8068258

[B147] Roe F. J. (1977). Metronidazole: Review of Uses and Toxicity. J. Antimicrob. Chemother. 3, 205–212. doi: 10.1093/jac/3.3.205 559669

[B148] Rossignol J. F. (2010). Cryptosporidium and Giardia: Treatment Options and Prospects for New Drugs. Exp. Parasitol. 124, 45–53. doi: 10.1016/j.exppara.2009.07.005 19632225

[B149] Saad H. A. Soliman M. I. Azzam A. M. Mostafa B. (2015). Antiparasitic Activity of Silver and Copper Oxide Nanoparticles Against Entamoeba Histolytica and Cryptosporidium Parvum Cysts. J. Egypt Soc. Parasitol. 45, 593–602. doi: 10.12816/0017920 26939237

[B150] Said D. E. Elsamad L. M. Gohar Y. M. (2012). Validity of Silver, Chitosan, and Curcumin Nanoparticles as Anti-Giardia Agents. Parasitol. Res. 111, 545–554. doi: 10.1007/s00436-012-2866-1 22392135

[B151] Satterwhite C. L. Torrone E. Meites E. Dunne E. F. Mahajan R. Ocfemia M. C.. (2013). Sexually Transmitted Infections Among US Women and Men: Prevalence and Incidence Estimates 2008. Sex Transm. Dis. 40, 187–193. doi: 10.1097/OLQ.0b013e318286bb53 23403598

[B152] Sauvey C. Ehrenkaufer G. Shi D. Debnath A. Abagyan R. (2021). Antineoplastic Kinase Inhibitors: A New Class of Potent Anti-Amoebic Compounds. PloS Negl. Trop. Dis. 15, e0008425. doi: 10.1371/journal.pntd.0008425 33556060 PMC7895358

[B153] Scallan E. Hoekstra R. M. Angulo F. J. Tauxe R. V. Widdowson M. A. Roy S. L.. (2011). Foodborne Illness Acquired in the United States–major Pathogens. Emerg. Infect. Dis. 17, 7–15. doi: 10.3201/eid1701.P11101 21192848 PMC3375761

[B154] Schmid G. Narcisi E. Mosure D. Secor W. E. Higgins J. Moreno H. (2001). Prevalence of Metronidazole-Resistant Trichomonas Vaginalis in a Gynecology Clinic. J. Reprod. Med. 46, 545–549. doi: 10.1097/00006254-200111000-00015 11441678

[B155] Schwebke J. R. Barrientes F. J. (2006). Prevalence of Trichomonas Vaginalis Isolates With Resistance to Metronidazole and Tinidazole. Antimicrob. Agents Chemother. 50, 4209–4210. doi: 10.1128/AAC.00814-06 17000740 PMC1693974

[B156] Secor W. E. Meites E. Starr M. C. Workowski K. A. (2014). Neglected Parasitic Infections in the United States: Trichomoniasis. Am. J. Trop. Med. Hyg. 90, 800–804. doi: 10.4269/ajtmh.13-0723 24808247 PMC4015567

[B157] Sedighi F. Abbasali P. R. Maghsood A. Fallah M. (2016). Comparison of Therapeutic Effect of Anti-Cryptosporidium Nano-Nitazoxanide (ntz) with Free Form of This Drug in Neonatal Rat. Scientific J. Hamadan Univ. Med. Sci. 23, 134–140.

[B158] Sena A. C. Bachmann L. H. Hobbs M. M. (2014). Persistent and Recurrent Trichomonas Vaginalis Infections: Epidemiology, Treatment and Management Considerations. Expert Rev. Anti Infect. Ther. 12, 673–685. doi: 10.1586/14787210.2014.887440 24555561

[B159] Sharma S. Ahuja V. (2021). Liver Abscess: Complications and Treatment. Clin. Liver Dis. (Hoboken) 18, 122–126. doi: 10.1002/cld.1128 34691398 PMC8518335

[B160] Sharma P. Khurana S. Sharma A. Sehgal R. Malla N. (2016). Presence of Intracellular Viruses in Human Cryptosporidium Isolates. Ann. Parasitol. 62, 139–147. doi: 10.17420/ap6202.46 27614480

[B161] Sherrard J. Wilson J. Donders G. Mendling W. Jensen J. S. (2018). 2018 European (IUSTI/WHO) International Union Against Sexually Transmitted Infections (IUSTI) World Health Organisation (WHO) Guideline on the Management of Vaginal Discharge. Int. J. STD AIDS 29, 1258–1272. doi: 10.1177/0956462418785451 30049258

[B162] Shirley D. T. Farr L. Watanabe K. Moonah S. (2018). A Review of the Global Burden, New Diagnostics, and Current Therapeutics for Amebiasis. Open Forum Infect. Dis. 5, ofy161. doi: 10.1093/ofid/ofy161 30046644 PMC6055529

[B163] Shirley D. A. Sharma I. Warren C. A. Moonah S. (2021). Drug Repurposing of the Alcohol Abuse Medication Disulfiram as an Anti-Parasitic Agent. Front. Cell Infect. Microbiol. 11, 633194. doi: 10.3389/fcimb.2021.633194 33777846 PMC7991622

[B164] Shrivastava A. K. Kumar S. Smith W. A. Sahu P. S. (2017). Revisiting the Global Problem of Cryptosporidiosis and Recommendations. Trop. Parasitol. 7, 8–17. doi: 10.4103/2229-5070.202290 28459010 PMC5369280

[B165] Shrivastav M. T. Malik Z. Somlata (2020). Revisiting Drug Development Against the Neglected Tropical Disease, Amebiasis. Front. Cell Infect. Microbiol. 10, 628257. doi: 10.3389/fcimb.2020.628257 33718258 PMC7943716

[B166] Silver B. J. Guy R. J. Kaldor J. M. Jamil M. S. Rumbold A. R. (2014). Trichomonas Vaginalis as a Cause of Perinatal Morbidity: A Systematic Review and Meta-Analysis. Sex Transm. Dis. 41, 369–376. doi: 10.1097/OLQ.0000000000000134 24825333

[B167] Sinkala E. Katubulushi M. Sianongo S. Obwaller A. Kelly P. (2011). In a Trial of the Use of Miltefosine to Treat HIV-Related Cryptosporidiosis in Zambian Adults, Extreme Metabolic Disturbances Contribute to High Mortality. Ann. Trop. Med. Parasitol. 105, 129–134. doi: 10.1179/136485911X12899838683160 21396249 PMC4084659

[B168] Sisson G. Jeong J. Y. Goodwin A. Bryden L. Rossler N. Lim-Morrison S.. (2000). Metronidazole Activation is Mutagenic and Causes DNA Fragmentation in Helicobacter Pylori and in Escherichia Coli Containing a Cloned H. Pylori RdxA(+) (Nitroreductase) Gene. J. Bacteriol. 182, 5091–5096. doi: 10.1128/JB.182.18.5091-5096.2000 10960092 PMC94656

[B169] Snipes L. J. Gamard P. M. Narcisi E. M. Beard C. B. Lehmann T. Secor W. E. (2000). Molecular Epidemiology of Metronidazole Resistance in a Population of Trichomonas Vaginalis Clinical Isolates. J. Clin. Microbiol. 38, 3004–3009. doi: 10.1128/JCM.38.8.3004-3009.2000 10921968 PMC87171

[B170] Soares F. A. Benitez A. D. N. Santos B. M. D. Loiola S. H. N. Rosa S. L. Nagata W. B.. (2020). A Historical Review of the Techniques of Recovery of Parasites for Their Detection in Human Stools. Rev. Soc. Bras. Med. Trop. 53, e20190535. doi: 10.1590/0037-8682-0535-2019 32491097 PMC7269538

[B171] Soria-Arteche O. Hernandez-Campos A. Yepez-Mulia L. Trejo-Soto P. J. Hernandez-Luis F. Gres-Molina J.. (2013). Synthesis and Antiprotozoal Activity of Nitazoxanide-N-Methylbenzimidazole Hybrids. Bioorg. Med. Chem. Lett. 23, 6838–6841. doi: 10.1016/j.bmcl.2013.10.011 24183540

[B172] Speich B. Marti H. Ame S. M. Ali S. M. Bogoch I. I. Utzinger J.. (2013). Prevalence of Intestinal Protozoa Infection Among School-Aged Children on Pemba Island, Tanzania, and Effect of Single-Dose Albendazole, Nitazoxanide and Albendazole-Nitazoxanide. Parasitol. Vectors 6, 3. doi: 10.1186/1756-3305-6-3 PMC3558385 23289920

[B173] Sun Y. Chen D. Pan Y. Qu W. Hao H. Wang X.. (2019). Nanoparticles for Antiparasitic Drug Delivery. Drug Delivery 26, 1206–1221. doi: 10.1080/10717544.2019.1692968 31746243 PMC6882479

[B174] Su H. M. Tai J. H. (1996). Genomic Organization and Sequence Conservation in Type I Trichomonas Vaginalis Viruses. Virology 222, 470–473. doi: 10.1006/viro.1996.0446 8806533

[B175] Tejman-Yarden N. Miyamoto Y. Leitsch D. Santini J. Debnath A. Gut J.. (2013). A Reprofiled Drug, Auranofin, is Effective Against Metronidazole-Resistant Giardia Lamblia. Antimicrob. Agents Chemother. 57, 2029–2035. doi: 10.1128/AAC.01675-12 23403423 PMC3632933

[B176] Townson S. M. Upcroft J. A. Upcroft P. (1996). Characterisation and Purification of Pyruvate:Ferredoxin Oxidoreductase From Giardia Duodenalis. Mol. Biochem. Parasitol. 79, 183–193. doi: 10.1016/0166-6851(96)02661-8 8855555

[B177] Upcroft J. Mitchell R. Chen N. Upcroft P. (1996). Albendazole Resistance in Giardia is Correlated With Cytoskeletal Changes But Not With a Mutation at Amino Acid 200 in Beta-Tubulin. Microb. Drug Resist. 2, 303–308. doi: 10.1089/mdr.1996.2.303 9158790

[B178] Upcroft J. A. Upcroft P. (1993). Drug Resistance and Giardia. Parasitol. Today 9, 187–190. doi: 10.1016/0169-4758(93)90144-5 15463750

[B179] Upcroft P. Upcroft J. A. (2001). Drug Targets and Mechanisms of Resistance in the Anaerobic Protozoa. Clin. Microbiol. Rev. 14, 150–164. doi: 10.1128/CMR.14.1.150-164.2001 11148007 PMC88967

[B180] Valdez C. A. Tripp J. C. Miyamoto Y. Kalisiak J. Hruz P. Andersen Y. S.. (2009). Synthesis and Electrochemistry of 2-Ethenyl and 2-Ethanyl Derivatives of 5-Nitroimidazole and Antimicrobial Activity Against Giardia Lamblia. J. Med. Chem. 52, 4038–4053. doi: 10.1021/jm900356n 19480409 PMC2766634

[B181] Vazini H. (2017). Anti-Trichomonas Vaginalis Activity of Nano Micana Cordifolia and Metronidazole: An *In Vitro* Study. J. Parasitol. Dis. 41, 1034–1039. doi: 10.1007/s12639-017-0930-6 PMC5660030 29114138

[B182] Victoria-Hernandez J. A. Ventura-Saucedo A. Lopez-Morones A. Martinez-Hernandez S. L. Medina-Rosales M. N. Munoz-Ortega M.. (2020). Case Report: Multiple and Atypical Amoebic Cerebral Abscesses Resistant to Treatment. BMC Infect. Dis. 20, 669. doi: 10.1186/s12879-020-05391-y 32928130 PMC7490879

[B183] Vivancos V. Gonzalez-Alvarez I. Bermejo M. Gonzalez-Alvarez M. (2018). Giardiasis: Characteristics, Pathogenesis and New Insights About Treatment. Curr. Top. Med. Chem. 18, 1287–1303. doi: 10.2174/1568026618666181002095314 30277155

[B184] Wang B. Castellanos-Gonzalez A. White A. C. Jr. (2020). Novel Drug Targets for Treatment of Cryptosporidiosis. Expert Opin. Ther. Targets 24, 915–922. doi: 10.1080/14728222.2020.1785432 32552166

[B185] Wang A. L. Wang C. C. (1986). Discovery of a Specific Double-Stranded RNA Virus in Giardia Lamblia. Mol. Biochem. Parasitol. 21, 269–276. doi: 10.1016/0166-6851(86)90132-5 3807947

[B186] Wassmann C. Hellberg A. Tannich E. Bruchhaus I. (1999). Metronidazole Resistance in the Protozoan Parasite Entamoeba Histolytica is Associated With Increased Expression of Iron-Containing Superoxide Dismutase and Peroxiredoxin and Decreased Expression of Ferredoxin 1 and Flavin Reductase. J. Biol. Chem. 274, 26051–26056. doi: 10.1074/jbc.274.37.26051 10473552

[B187] Weir C. B. Le J. K. (2021). “Metronidazole,” in StatPearls (Treasure Island (FL): StatPearlsPublishing). Available at: http://www.ncbi.nlm.nih.gov/books/NB.

[B188] Workowski K. A. Bachmann L. H. Chan P. A. Johnston C. M. Muzny C. A. Park I.. (2021). Sexually Transmitted Infections Treatment Guideline. MMWR Recomm. Rep. 70, 1–187. doi: 10.15585/mmwr.rr7004a1 PMC8344968 34292926

[B189] Yang S. Zhao W. Wang H. Wang Y. Li J. Wu X. (2018). Trichomonas Vaginalis Infection-Associated Risk of Cervical Cancer: A Meta-Analysis. Eur. J. Obstet. Gynecol. Reprod. Biol. 228, 166–173. doi: 10.1016/j.ejogrb.2018.06.031 29980111

[B190] Zhang Y. Feng J. Mcmanus S. A. Lu H. D. Ristroph K. D. Cho E. J.. (2017). Design and Solidification of Fast-Releasing Clofazimine Nanoparticles for Treatment of Cryptosporidiosis. Mol. Pharm. 14, 3480–3488. doi: 10.1021/acs.molpharmaceut.7b00521 28929769 PMC5627342

