# Supplementary material for: An Overview of Mucosa-Associated Protozoa: Challenges in Chemotherapy and Future Perspectives
Source: Front Cell Infect Microbiol. 2022 Apr 25;12:860442. doi: 10.3389/fcimb.2022.860442 (PMC9084232; doi:10.3389/fcimb.2022.860442)
Supplement: Supplementary Table 1 — List of natural compounds with antiprotozoa activity. [file Table_1.pdf]

Supplementary table 1: List of natural compounds with antiprotozoa activity

Source	Compound	Activity	References
**Anti-giardiasis activities**			
Glycosmis trichanthera (leaves)	Methylgerambullin	EC_{50} = 14.6 µM (6.14 µg/mL)	(Drinic et al., 2019)
Geranium mexicanum	(–)-epicatechin, organic fraction and crude extract	IC_{50} 1.6-100.4 µg/mL	(Calzada et al., 2005)
Sambucus ebulus	Crude extract	IC_{50} 43 mg/mL	(Rahimi-Esboei et al., 2013)
Teloxys graveolens	Melilotoside	IC_{50} 16.8 µg/mL	(Calzada et al., 2003)
Rhamnus cathartica	Chitosan	10 µg/mL	(Yarahmadi et al., 2016)
		EC_{50} = 24mg/mL	(Chabra et al., 2019)
Rhamnus cathartica	Emodim	10 - 100 µg/kg	Chabra A et al., 2019)
**Anti-amebiasis activities**			
Glycosmis trichanthera (leaves)	methylgerambullin	EC_{50} = 14.5 µM (6.08 µg/ml)	(Drinić et al., 2019)
Geranium mexicanum	(–)-epicatechin	IC_{50} 1.9 µg/mL	(Calzada et al., 2005)
Helianthemum glomeratum	(–)-epigallocatechin	IC_{50} 6.89 µg/mL	(Meckes et al., 1999)
Osyris alba	(+)-catechin	IC_{50} 17.67 µg/mL	(Al-Jaber et al., 2010)
Helianthemum glomeratum	Kaempferol	IC_{50} 27.7 µM	(Bolanos et al., 2015)
Morinda morindoides		IC_{50} 10.3 µg/mL	(Cimanga et al., 2006)
Morinda morindoides	Apigenin	IC_{50} 10.06 µg/mL	(Cimanga et al., 2006)
Helianthemum glomeratum	Geranin A	IC_{50} 184.7 µg/mL	(Meckes et al., 1999)
	Geranin B	IC_{50} 13.6 µg/mL	(Meckes et al., 1999)
Geranium niveum	Geraldin C	IC_{50} 52 µg/mL	(Calzada et al., 2001)
	Geranin D	IC_{50} 28.6 µg/mL	(Calzada et al., 2001)
Helianthemum glomeratum	Isoquercitrin	IC_{50} 14.7 µg/mL	(Calzada and Alanis, 2007)
Sphaeralcea angustifolia	Tiliroside	IC_{50} 17.5 µg/mL	(Calzada et al., 2017)
Morinda morindoides	Luteolin	IC_{50} 17.8 µg/mL	(Cimanga et al., 2006)
Osyris alba	(+)-catechin-3-O-α L-rhamnopyranoside	IC_{50} 29.67 µg/mL	(Al-Jaber et al., 2010)

Morinda morindoides	Quercetin	IC_{50} 114.30 µg/mL	(Cimanga et al., 2006)
Teloxys graveolens	Melilotoside	IC_{50}12.5 µg/mL	(Calzada et al., 2003)
Grape skin and other food products	Resveratrol (trans-3,4',5-trihydroxystilbene)	IC_{50} 220µM	(Pais-Morales et al., 2016)
**Anti-cryptosporidiasis activities**			
Streptomyces	Mitomycin	EC_{50} =0.133 µM	(Jin et al., 2019)
Aspergillus terrous	Lovastatin	EC_{50} = 2.406 µM	(Jin et al., 2019)
Streptomyces	Daunorubicin	EC_{50} = 1.494 µM	(Jin et al., 2019)
Streptomyces	Valinomycin	EC_{50} =0.122 µM	(Jin et al., 2019)
Streptomyces	Dactinomycin	EC_{50} = 0.314 µM	(Jin et al., 2019)
Toona ciliate	Cedrelone	EC_{50} = 0.267 µM	(Jin et al., 2019)
Biancaea sappan	Deoxysappanone B 7,4´ – dimethyl ether	EC_{50} = 0.734 µM	(Jin et al., 2019)
Biancaea sappan	Deoxysappanone B 7,3´ – dimethyl ether	EC_{50} = 1.187 µM	(Jin et al., 2019)
Salvia miltiorrhiza	Tanshinone IIA	EC_{50} = 0.964 µM	(Jin et al., 2019)
Scutellaria baicalensis	Baicalein	EC_{50} = 0.981 µM	(Jin et al., 2019)
Garcinia hanburyi	Dihydrogambogic acid	EC_{50} = 1.669 µM	(Jin et al., 2019)
Azadirachta indica	Deacetylgedunin	EC_{50} = 1.771 µM	(Jin et al., 2019)
Carapa guianensis	Deacetoxy-7-oxogedunin	EC_{50} = 1.943 µM	(Jin et al., 2019)
**Anti-trichomonad activities**			
Amomum tsa-ko	Geracinol	171-343µg/mL	(Dai et al., 2016)
Nectandra megapotamica	(+) α-Bisabolol	IC_{50} 98.7µM	(Farias et al., 2019)
Vitis vinifera (grapes)	Resveratrol	IC_{50} 25µM	(Mallo et al., 2013)
Lycopersicon esculentum	Tomatidine	3.2-22.9%	(Liu et al., 2016)
Lycopersicon esculentum	Tomatine	IC_{50} 2.0-7.9µM	(Liu et al., 2016)
Cassnia holstii	Hedargenin	IC_{50} 2.8µM	(Mehriardestani et al., 2017)
Hypericum spp.	Isoaustrobrasilol B	IC_{50} 38 µm	(Menezes et al., 2017)
Palatanus acerifoli	Betulinic acid	MIC 91.2 µM	(Innocente et al., 2014)

Hypericum polyanthenum	Benzopyrans	damage to the parasites membrane	(Cargnin et al., 2013)
Sapindus saponaria	Saponin A	MIC 0.025%;	(Rocha et al., 2012)
Sapindus saponaria	Saponin B	MIC 0.16mg/mL	(Damke et al., 2013)
Solanum torvum	Torvosides	MIC 6.2 -12.5 μM	(Arthan et al., 2008)
Hypericum polyanthenum	Uliginosin B	damage to the parasites membrane	(Cargnin et al., 2013)
Manika rufula	Ursolic acid	MIC 25 μM	(Bitencourt et al., 2018)
Hippeastrum morelianum	Candimine	Cell demage	(Giordani et al., 2010)
Hippeastrum morelianum	Lycorine	Cell demage	(Giordani et al., 2010)
Hippeastrum breviflorum	Lycorine	Cell demage	(Vieira Pde et al., 2011)
Hippeastrum breviflorum	Lycosinine	Cell demage	(Vieira Pde et al., 2011)
Rheum palmatum	Emodin	inhibitory effect on the pathogenicity in murine models	(Wang, 1993)
Solanum tuberosum (potatoes)	caffeic acid	21.1-42.8% inhibition	(O'Donoghue et al., 2019)
Solanum tuberosum (potatoes)	α-chaconine	IC_{50} 35-60 μM	(O'Donoghue et al., 2019)
Solanum tuberosum (potatoes)	chlorogenic acid	11.4-21.9% inhibition	(O'Donoghue et al., 2019)
Solanum tuberosum (potatoes)	quercetin	18.5-46.6% inhibition	(O'Donoghue et al., 2019)
Solanum tuberosum (potatoes)	solanidine	22.6-48.4% inhibition	(O'Donoghue et al., 2019)
Solanum tuberosum (potatoes)	α- solanidine	IC_{50} 10.9-16.8 μM	(O'Donoghue et al., 2019)
Phaseolus vulgaris (kidney beans)	Lectin	Ultrastructure cell damage	(Aminou et al., 2016)
Proteosome inhibitor	Carmaphycin-17	reduced parasite burden in murine model	(O'Donoghue et al., 2019)
Plant hormone	Methyl jasmonate	Cell dealth	(Ofer et al., 2008)

Morinda panamensis (plant roots)	Lucidin-isopropyl-ether	IC_{50} 1.32 µg/mL	(Caceres-Castillo et al., 2019)
Fugal endophyte E6927E	Pyrrolocin A	EC_{50} 60nM	(King et al., 2019)
Scutellaria havanensis (plant leaves)	Wogonine	IC_{50} 7.7-32.2 µg/mL	(Fernandez-Calienes Valdes et al., 2016)
*Argemone mexican (*flowering thistle)	Stem and leaf extract	IC_{50} 70.8 and 67.2 µg/mL	(Hashemi et al., 2021)

References

AL-JABER, H. I., MOSLEH, I. M., MALLOUH, A., ABU SALIM, O. M. & ABU ZARGA, M. H. 2010. Chemical constituents of Osyris alba and their antiparasitic activities. *J Asian Nat Prod Res,* 12**,** 814-20. doi:10.1080/10286020.2010.502892.

AMINOU, H. A., ALAM-ELDIN, Y. H. & HASHEM, H. A. 2016. Effect of Nigella sativa alcoholic extract and oil, as well as Phaseolus vulgaris (kidney bean) lectin on the ultrastructure of Trichomonas vaginalis trophozoites. *J Parasit Dis,* 40**,** 707-13. doi:10.1007/s12639-014-0564-x.

ARTHAN, D., SITHIPROM, S., THIMA, K., LIMMATVATIRAT, C., CHAVALITSHEWINKOON-PETMITR, P. & SVASTI, J. 2008. Inhibitory effects of Thai plants beta-glycosides on Trichomonas vaginalis. *Parasitol Res,* 103**,** 443-8. doi:10.1007/s00436-008-0996-2.

BITENCOURT, F. G., DE BRUM VIEIRA, P., MEIRELLES, L. C., RIGO, G. V., DA SILVA, E. F., GNOATTO, S. C. B. & TASCA, T. 2018. Anti-Trichomonas vaginalis activity of ursolic acid derivative: a promising alternative. *Parasitol Res,* 117**,** 1573-1580. doi: 10.1007/s00436-018-5839-1.

BOLANOS, V., DIAZ-MARTINEZ, A., SOTO, J., MARCHAT, L. A., SANCHEZ-MONROY,V. & RAMIREZ-MORENO, E. 2015. Kaempferol inhibits Entamoeba histolytica growth by altering cytoskeletal functions. *Mol Biochem Parasitol,* 204**,** 16-25. doi:10.1016/j.molbiopara.2015.11.004.

CACERES-CASTILLO, D., PEREZ-NAVARRO, Y., TORRES-ROMERO, J. C., MIRON-LOPEZ, G., CEBALLOS-CRUZ, J., ARANA-ARGAEZ, V., VAZQUEZ-CARRILLO, L., FERNANDEZ-SANCHEZ, J. M. & ALVAREZ-SANCHEZ, M. E. 2019. Trichomonicidal activity of a new anthraquinone isolated from the roots of Morinda panamensis Seem. *Drug Dev Res,* 80**,** 155-161. doi:10.1002/ddr.21504.

CALZADA, F. & ALANIS, A. D. 2007. Additional antiprotozoal flavonol glycosides of the aerial parts of Helianthemum glomeratum. *Phytother Res,* 21**,** 78-80. doi:10.1002/ptr.2031.

CALZADA, F., BASURTO, J. C., BARBOSA, E., VELAZQUEZ, C., HERNANDEZ, N. G., ORDONEZ RAZO, R. M., LUNA, D. M. & MULIA, L. Y. 2017. Antiprotozoal

Activities of Tiliroside and other Compounds from Sphaeralcea angustifolia (Cav.) G. Don. *Pharmacognosy Res,* 9**,** 133-137. doi:10.4103/0974-8490.204644.

CALZADA, F., CEDILLO-RIVERA, R., BYE, R. & MATA, R. 2001. Geranins C and D, additional new antiprotozoal A-type proanthocyanidins from Geranium niveum. *Planta Med,* 67**,** 677-80. Doi:10.1055/s-2001-17358.

CALZADA, F., CERVANTES-MARTINEZ, J. A. & YEPEZ-MULIA, L. 2005. In vitro antiprotozoal activity from the roots of Geranium mexicanum and its constituents on Entamoeba histolytica and Giardia lamblia. *J Ethnopharmacol,* 98**,** 191-3. doi:10.1016/j.jep.2005.01.019.

CALZADA, F., VELAZQUEZ, C., CEDILLO-RIVERA, R. & ESQUIVEL, B. 2003. Antiprotozoal activity of the constituents of Teloxys graveolens. *Phytother Res,* 17**,** 731-2. doi:10.1002/ptr.1192.

CARGNIN, S. T., VIEIRA PDE, B., CIBULSKI, S., CASSEL, E., VARGAS, R. M., MONTANHA, J., ROEHE, P., TASCA, T. & VON POSER, G. L. 2013. Anti-Trichomonas vaginalis activity of Hypericum polyanthemum extract obtained by supercritical fluid extraction and isolated compounds. *Parasitol Int,* 62**,** 112-7. doi:10.1016/j.parint.2012.10.006.

CHABRA, A., RAHIMI-ESBOEI, B., HABIBI, E., MONADI, T., AZADBAKHT, M., ELMI, T., VALIAN, H. K., AKHTARI, J., FAKHAR, M. & NAGHSHVAR, F. 2019. Effects of some natural products from fungal and herbal sources on Giardia lamblia in vivo. *Parasitology,* 146**,** 1188-1198. doi:10.1017/S0031182019000325.

CIMANGA, R. K., KAMBU, K., TONA, L., HERMANS, N., APERS, S., TOTTE, J., PIETERS, L. & VLIETINCK, A. J. 2006. Cytotoxicity and in vitro susceptibility of Entamoeba histolytica to Morinda morindoides leaf extracts and its isolated constituents. *J Ethnopharmacol,* 107**,** 83-90. doi:10.1016/j.jep.2006.02.010.

DAI, M., PENG, C., PENG, F., XIE, C., WANG, P. & SUN, F. 2016. Anti-Trichomonas vaginalis properties of the oil of Amomum tsao-ko and its major component, geraniol. *Pharm Biol,* 54**,** 445-50. doi:10.3109/13880209.2015.1044617.

DAMKE, E., TSUZUKI, J. K., CHASSOT, F., CORTEZ, D. A., FERREIRA, I. C., MESQUITA, C. S., DA-SILVA, V. R., SVIDZINSKI, T. I. & CONSOLARO, M. E. 2013. Spermicidal and anti-Trichomonas vaginalis activity of Brazilian Sapindus saponaria. *BMC Complement Altern Med,* 13**,** 196. doi:10.1186/1472-6882-13-196.

DRINIC, M., RANINGER, A., ZRAUNIG, A., ASTELBAUER, F., LEITSCH, D., OBWALLER, A., WALOCHNIK, J., GREGER, H. & DUCHENE, M. 2019. Activity of methylgerambullin from Glycosmis species (Rutaceae) against *Entamoeba histolytica* and *Giardia duodenalis* in vitro. *Int J Parasitol Drugs Drug Resist,* 10**,** 109-117. doi:10.1016/j.ijpddr.2019.08.001.

FARIAS, K. S., KATO, N. N., BOARETTO, A. G., WEBER, J. I., BRUST, F. R., ALVES, F. M., TASCA, T., MACEDO, A. J., SILVA, D. B. & CAROLLO, C. A. 2019. Nectandra as a renewable source for (+)-alpha-bisabolol, an antibiofilm and anti-Trichomonas vaginalis compound. *Fitoterapia,* 136**,** 104179. doi:10.1016/j.fitote.2019.104179.

FERNANDEZ-CALIENES VALDES, A., MONZOTE FIDALGO, L., SARIEGO RAMOS, I., MARRERO DELANGE, D., MORALES RICO, C. L., MENDIOLA MARTINEZ, J. &

CUELLAR, A. C. 2016. Antiprotozoal screening of the Cuban native plant Scutellaria havanensis. *Pharm Biol,* 54**,** 3197-3202. doi:10.1080/13880209.2016.1216130.

GIORDANI, R. B., WEIZENMANN, M., ROSEMBERG, D. B., DE CARLI, G. A., BOGO, M. R., ZUANAZZI, J. A. & TASCA, T. 2010. Trichomonas vaginalis nucleoside triphosphate diphosphohydrolase and ecto-5'-nucleotidase activities are inhibited by lycorine and candimine. *Parasitol Int,* 59**,** 226-31. doi:10.1016/j.parint.2010.02.004.

HASHEMI, N., OMMI, D., KHEYRI, P., KHAMESIPOUR, F., SETZER, W. N. & BENCHIMOL, M. 2021. A review study on the anti-trichomonas activities of medicinal plants. *Int J Parasitol Drugs Drug Resist,* 15**,** 92-104. doi:10.1016/j.ijpddr.2021.01.002.

INNOCENTE, A. M., VIEIRA PDE, B., FRASSON, A. P., CASANOVA, B. B., GOSMANN, G., GNOATTO, S. C. & TASCA, T. 2014. Anti-Trichomonas vaginalis activity from triterpenoid derivatives. *Parasitol Res,* 113**,** 2933-40. doi:10.1007/s00436-014-3955-0.

JIN, Z., MA, J., ZHU, G. & ZHANG, H. 2019. Discovery of Novel Anti-cryptosporidial Activities From Natural Products by in vitro High-Throughput Phenotypic Screening. *Front Microbiol,* 10**,** 1999. doi:10.3389/fmicb.2019.01999.

KING, J. B., CARTER, A. C., DAI, W., LEE, J. W., KIL, Y. S., DU, L., HELFF, S. K., CAI, S., HUDDLE, B. C. & CICHEWICZ, R. H. 2019. Design and Application of a High-Throughput, High-Content Screening System for Natural Product Inhibitors of the Human Parasite Trichomonas vaginalis. *ACS Infect Dis,* 5**,** 1456-1470. doi:10.1021/acsinfecdis.9b00156.

LIU, J., KANETAKE, S., WU, Y. H., TAM, C., CHENG, L. W., LAND, K. M. & FRIEDMAN, M. 2016. Antiprotozoal Effects of the Tomato Tetrasaccharide Glycoalkaloid Tomatine and the Aglycone Tomatidine on Mucosal Trichomonads. *J Agric Food Chem,* 64**,** 8806-8810. doi:10.1021/acs.jafc.6b04030.

MALLO, N., LAMAS, J. & LEIRO, J. M. 2013. Hydrogenosome metabolism is the key target for antiparasitic activity of resveratrol against Trichomonas vaginalis. *Antimicrob Agents Chemother,* 57**,** 2476-84. doi:10.1128/AAC.00009-13.

MECKES, M., CALZADA, F., TAPIA-CONTRERAS, A. & CEDILLO-RIVERA, R. 1999. Antiprotozoal properties of Helianthemum glomeratum. *Phytother Res,* 13**,** 102-5.

MEHRIARDESTANI, M., ALIAHMADI, A., TOLIAT, T. & RAHIMI, R. 2017. Medicinal plants and their isolated compounds showing anti-Trichomonas vaginalis- activity. *Biomed Pharmacother,* 88**,** 885-893. doi:10.1016/j.biopha.2017.01.149.

MENEZES, C. B., RIGO, G. V., BRIDI, H., TRENTIN, D. D. S., MACEDO, A. J., VON POSER, G. L. & TASCA, T. 2017. The anti-Trichomonas vaginalis phloroglucinol derivative isoaustrobrasilol B modulates extracellular nucleotide hydrolysis. *Chem Biol Drug Des,* 90**,** 811-819. doi:10.1111/cbdd.13002.

OFER, K., GOLD, D. & FLESCHER, E. 2008. Methyl jasmonate induces cell cycle block and cell death in the amitochondriate parasite Trichomonas vaginalis. *Int J Parasitol,* 38**,** 959-68. doi:10.1016/j.ijpara.2007.12.008.

PAIS-MORALES, J., BETANZOS, A., GARCIA-RIVERA, G., CHAVEZ-MUNGUIA, B., SHIBAYAMA, M. & OROZCO, E. 2016. Resveratrol Induces Apoptosis-Like Death

and Prevents In Vitro and In Vivo Virulence of Entamoeba histolytica. *PLoS One,* 11**,** e0146287. doi:10.1371/journal.pone.0146287.

RAHIMI-ESBOEI, B., EBRAHIMZADEH, M. A., GHOLAMI, S. & FALAH-OMRANI, V. 2013. Anti-giardial activity of Sambucus ebulus. *Eur Rev Med Pharmacol Sci,* 17**,** 2047-50. PMID: 23884825.

ROCHA, T. D., DE BRUM VIEIRA, P., GNOATTO, S. C., TASCA, T. & GOSMANN, G. 2012. Anti-Trichomonas vaginalis activity of saponins from Quillaja, Passiflora, and Ilex species. *Parasitol Res,* 110**,** 2551-6. doi:10.1007/s00436-011-2798-1.

VIEIRA PDE, B., GIORDANI, R. B., DE CARLI, G. A., ZUANAZZI, J. A. & TASCA, T. 2011. Screening and bioguided fractionation of Amaryllidaceae species with anti-Trichomonas vaginalis activity. *Planta Med,* 77**,** 1054-9. doi:10.1055/s-0030-1270740.

WANG, H. H. 1993. Antitrichomonal action of emodin in mice. *J Ethnopharmacol,* 40**,** 111-6. doi:10.1016/0378-8741(93)90055-a.

YARAHMADI, M., FAKHAR, M., EBRAHIMZADEH, M. A., CHABRA, A. & RAHIMI-ESBOEI, B. 2016. The anti-giardial effectiveness of fungal and commercial chitosan against Giardia intestinalis cysts in vitro. *J Parasit Dis,* 40**,** 75-80. doi:10.1007/s12639-014-0449-z.
